# Orbitofrontal cortex microRNAs support long-lasting heroin seeking behavior in male rats

**DOI:** 10.1038/s41398-023-02423-4

**Published:** 2023-04-08

**Authors:** Mary Tresa Zanda, Gabriele Floris, Stephanie E. Daws

**Affiliations:** 1 Center for Substance Abuse Research, Temple University, Philadelphia, PA USA; 2 Department of Neural Sciences, Temple University, Philadelphia, PA USA

**Keywords:** Molecular neuroscience, Addiction

## Abstract

Recovery from opioid use disorder (OUD) and maintenance of abstinence from opioid use is hampered by perseverant drug cravings that may persist for months after cessation of drug use. Drug cravings can intensify during the abstinence period, a phenomenon referred to as the ‘incubation of craving’ that has been well-described in preclinical studies. We previously reported that animals that self-administered heroin at a dosage of 0.075 mg/kg/infusion (HH) paired with discrete drug cues displayed robust incubation of heroin craving behavior after 21 days (D) of forced abstinence, an effect that was not observed with a lower dosage (0.03 mg/kg/infusion; HL). Here, we sought to elucidate molecular mechanisms underlying long-term heroin seeking behavior by profiling microRNA (miRNA) pathways in the orbitofrontal cortex (OFC), a brain region that modulates incubation of heroin seeking. miRNAs are small noncoding RNAs with long half-lives that have emerged as critical regulators of drug seeking behavior but their expression in the OFC has not been examined in any drug exposure paradigm. We employed next generation sequencing to detect OFC miRNAs differentially expressed after 21D of forced abstinence between HH and HL animals, and proteomics analysis to elucidate miRNA-dependent translational neuroadaptations. We identified 55 OFC miRNAs associated with incubation of heroin craving, including miR-485-5p, which was significantly downregulated following 21D forced abstinence in HH but not HL animals. We bidirectionally manipulated miR-485-5p in the OFC to demonstrate that miR-485-5p can regulate long-lasting heroin seeking behavior after extended forced abstinence. Proteomics analysis identified 45 proteins selectively regulated in the OFC of HH but not HL animals that underwent 21D forced abstinence, of which 7 were putative miR-485-5p target genes. Thus, the miR-485-5p pathway is dysregulated in animals with a phenotype of persistent heroin craving behavior and OFC miR-485-5p pathways may function to support long-lasting heroin seeking.

## Introduction

Opioid use disorder (OUD) is a chronically relapsing disorder, characterized by compulsive drug use and lack of control in restricting drug intake, accompanied by dysphoric behavior when access to the drug is precluded [1]. One of the critical limitations hampering recovery from OUD is lack of effective treatments. While current FDA-approved medications can treat withdrawal symptoms associated with opioid use cessation and substitute for misused opioids, the relapse rate remains high, with ~57–65% of OUD patients experiencing relapse after weeks of stabilization with medications [2]. In humans, environmental context as well as cues paired to the intake of the highly addictive opioid heroin play a critical role in the consolidation of associated learning of the conditioned-reinforcement properties of the drug [3, 4]. Such conditions contribute to not only drug-seeking behavior, but also relapse after long periods of abstinence [5]. Indeed, prior studies have demonstrated that cue-induced drug-seeking progressively increases during abstinence, a phenomenon known as the “incubation of drug craving” [6]. There are three main events that trigger relapse: first, the priming effect of the drug [7]; second, the presentation of cues previously associated with drug intake [8, 9] and finally, stressful events, including negative symptoms associated with withdrawal [5, 10, 11]. Relapse behavior is extensively studied using preclinical rodent models. In forced abstinence [12] and incubation of drug craving [13] models, animals remain in their homecage with no extinction. After a period of time, animals are re-exposed to the same context previously paired with the drug self-administration, and the drug-seeking is assessed by non-reinforced lever presses [14]. Previous research from our laboratory has demonstrated that the incubation of heroin craving after 21 days of forced abstinence requires a unique combination of discrete drug cues with drug dosage [15]. Animals that self-administered heroin at a dosage of 0.075 mg/kg/infusion paired with discrete drug cues displayed robust incubation behavior, an effect that was not observed with only discriminative drug cues or a lower dosage of 0.03 mg/kg/infusion, despite controlling for total drug intake [15]. The goal of the present study was to use such divergent behavioral phenotypes as a tool to: 1) to elucidate the molecular mechanisms associated with heroin incubation behavior; and 2) to identify molecular determinants that modulate long-lasting heroin seeking.

While chronic drug use results in neuroadaptations that contribute to its long-lasting nature [16], molecular mechanisms of relapse are still poorly understood. Such drug exposure results in alterations in gene expression [17–19] and protein expression [19, 20], as well as effects that are independent from transcription such as reduced protein degradation or receptor sensitivity [21–24] and structural changes at the level of the spine density [5, 25–27]. In general, it is assumed that relapse is supported by similar types of neural mechanisms [28], but many of the precise molecular and cellular players are yet to be elucidated. These aforementioned drug-induced neuroplasticity modifications are finely regulated by a class of small non-coding RNA, named microRNAs (miRNAs). miRNAs are considered “master regulators” of gene expression, due to the fact that they control targeted mRNA fate at the post-transcriptional level [29, 30]. miRNAs are sequences of ~22 nucleotides that bind to the 3′ untranslated regions (UTRs) in target mRNAs with sequence complementarity at a ‘seed region’ [31, 32]. Binding between targeted mRNA and miRNAs is regulated by the RNA-induced silencing complex (RISC), which results in translational repression or mRNA cleavage according to the level of mRNA-miRNA complementarity [31, 32].

Researchers in the drug addiction field have studied the relationship between drugs and miRNAs for different class of drugs. Exposure to psychostimulants, alcohol, nicotine, or opioids in preclinical models results in unique miRNA expression patterns that may vary according to brain region [32–58]. Work with human patient samples has also reported regulation of miRNA expression in macrophages after morphine stimulation and in the serum of heroin-dependent patients [56, 58, 59]. Such studies demonstrate that regulation of miRNAs is a translationally-relevant, drug-induced consequence. Moreover, manipulation of miRNA expression can regulate opioid-seeking behavior in rodent models of conditioned place preference and self-administration [60–62]. However, the role of miRNAs in perpetuating long-lasting opioid seeking behavior is currently unknown. Two studies have explored the duration of miRNA regulation following opioid exposure and reported that miRNA regulation is a lasting consequence of morphine conditioned placed preference and heroin self-administration [60, 61]. Indeed, miRNAs have a long half-life and can regulate remote memory in other preclinical models [63, 64]. In this study, we hypothesized that heroin self-administration results in long-lasting regulation of miRNAs in the orbitofrontal cortex (OFC). The OFC, located within the frontal cortex, mediates long-lasting seeking for opioids after forced abstinence in rodent models of the incubation of heroin craving [65–67]. Moreover, human subjects that chronically use heroin have increased blood flow into the OFC when experiencing drug craving [68]. Such perseverant regulation of OFC miRNAs may support incubation of heroin seeking and long-lasting heroin seeking behavior. By employing an unbiased omics approach, we identified a pattern of OFC miRNAs and putative protein targets selectively regulated after 21D of abstinence from the high heroin dosage but not the low. Furthermore, we performed functional manipulations in the OFC to demonstrate that the incubation-associated miRNA miR-485-5p is capable of bidirectionally regulating long-lasting heroin seeking behavior. Thus, regulation of miRNA expression in the OFC is a long-lasting neuroadaptation of heroin exposure and may function to support perseverative heroin seeking behavior.

## Methods *(See supplemental information for additional detail)*

### Subjects

One hundred and forty-four adult male Sprague Dawley rats (Charles River Laboratories), 230–250 grams and aged 7 to 8 weeks old on arrival were used for this study. Rats were pair-housed on a reverse 12- hour light/dark cycle (lights off at 9:00 AM) with constant room temperature (22 ± 2°C) and humidity (40%) and provided with free access to laboratory chow and water. Animals were acclimated to the animal facility for 5–7 days before inclusion in the study. All procedures followed the National Institutes of Health’s Guide for the Care and Use of Laboratory Animals and were approved by Temple University’s Institutional Animal Care and Use Committee.

### Reagents

Heroin hydrochloride was supplied by the National Institute on Drug Abuse drug supply program and was dissolved in 0.9% sterile sodium chloride at a dosage of 0.075 mg/kg/infusion (high heroin, HH) or 0.03 mg/kg/infusion (low heroin, HL). miRIDIAN microRNA Rat rno-miR-485-5p mimic, miRIDIAN microRNA Rat rno-miR-485-5p hairpin inhibitor, miRIDIAN microRNA mimic Negative control #1 and miRIDIAN microRNA Hairpin Inhibitor Negative Control #1 were purchased from Horizon Discovery Ltd. (Lafayette, Colorado, USA) and administered at a concentration of 5 ug/µl. Negative control sequences are based on *C.elegans* microRNA (miR-67) with minimal sequence identity with miRNAs in rat. For validation of mimics and inhibitors, *n* = 4/group. For in vivo delivery of mimics after 21D forced abstinence, CTRL mimic *n* = 8; miR-485-4p *n* = 11. For in vivo delivery of inhibitors after 2D forced abstinence, *n* = 8/group; after 21D forced abstinence, CTRL inhibitor, *n* = 9; miR485-5p inhibitor, *n* = 8. scAAV9-EF1a-rno-mir-485-eGFP and scAAV9-EF1a-ctrl-miR-eGFP, purchased from Vector Biolabs (Malvern, Pennsylvania, USA), were packed into AAV9 serotype, under an EF1a promoter with an eGFP reporter. The viral titer levers were 1.4 × 10^13^ GC/mL for scAAV9-EF1a-rno-mir-485-eGFP and 1.5 × 10^13^ GC/mL for scAAV0-EF1a-ctrl-miR-eGFP. For validation of viral overexpression of miR-485-5p at 10D, *n* = 4/group; at 15D, *n* = 5/group. For in vivo delivery of viruses after 21D forced abstinence, CTRL virus *n* = 15; miR-485-5p virus *n* = 16.

### Self-administration procedure, relapse test and OFC infusion

Drug self-administration studies were conducted in Skinner boxes under a fixed ratio (FR) 1 schedule of reinforcement for 10 days in 6-hour daily sessions using the discrete cues protocol as previously described [15]. Following 10 days of heroin self-administration, animals underwent 2 or 21D forced abstinence in their homecage (*n* = 12/group) and were then euthanized for molecular analysis. Infusion of miRNA mimics, inhibitors, or viruses into cannula during the forced abstinence period occurred in animals from additional cohorts (see animals numbers in ‘Reagents’ section) using 30-gauge internal injectors that projected 1 mm below the guide cannula, attached with PE-20 tubing to 10 µl Hamilton syringes using a multichannel pump (KD Scientific Inc., Holliston, Massachusetts, USA) over 5 min at a rate of 3.33 nl/second.

### Tissue collection and extraction of RNA or protein

For extraction of total RNA or protein from OFC tissue samples, the miRVANA PARIS RNA extraction kit (Life Technologies, Carlsbad, CA) was used following the manufacturer’s instructions, as previously reported [64]. OFC was collected from the ventral (VO) and lateral subregions (LO). The RNA fraction was suspended in RNase free water and the RNA concentration was measured with a Qubit 3.0 Fluorometer and the Qubit RNA High Sensitivity Assay (Invitrogen, Carlsbad, California, USA). Protein fractions were suspended in 1X RIPA buffer (5X RIPA buffer: 100 mM Tris-HCl, 750 mM NaCl, 5 mM ethylenediaminetetraacetic acid [EDTA], 5 mM ethylene glycol-bis[β-aminoethyl ether]-N,N,N’,N’-tetraacetic acid [EGTA], 5% sodium deoxycholate, 0.5% sodium dodecyl sulfate [SDS]) and 1X Protease and Phosphatase Inhibitor Cocktail (Thermo Scientific).

### miRNA library preparation, sequencing, and data analysis

Small-RNA sequencing was performed on four biological replicates per group by BGI Genomics (BGI Americas Corp, Cambridge, MA, USA). DEseq2 [69] was used to calculate differentially expressed miRNAs with an adjusted p value of <0.05 and absolute value of Log2ratio of >1 considered statistically significant. DIANA, miRDB and TargetScan were used for target gene prediction of differentially expressed miRNAs. Gene Ontology and KEGG analysis for gene functional classification was performed using DAVID (NIH) [70, 71]. Raw sequencing data are available in the GEO repository (Accession # GSE205451). A list of log2 fold change and p-values for all miRNA profiling data can be found in Supplemental excel tables SE1, 2 and 3.

### Proteomic analysis

Proteomic analysis was performed on three biological replicates per group by the Core Research Facility at Yale University. OFC tissue punches were suspended in RIPA buffer containing protease inhibitor cocktail, homogenized, and prepared for the mass spectrometer read. Data were processed and analyzed using Proteome Discoverer (v.2.1; ThermoFisher Scientific) for MS and MS/MS peak picking and Mascot algorithm was used (version 2.6.1) for database searching. Results were considered when 2 unique peptides matched the same protein based on MOWSE score. MASCOT results were then uploaded to Scaffold (Proteome Software, Inc.) and unpaired t-tests were used to determine statistical significance between groups with *p* < 0.05 considered statistically significant. Data is available in Supplemental excel tables SE4, 5 and 6.

### miRNA and mRNA qPCR

For qPCR measurement of miRNA expression, 50 ng of total RNA was reverse transcribed into cDNA using the miRCURY LNA RT Kit (Qiagen, Germantown, MD) according to the manufacturer’s instructions, as previously described [72]. Expression levels were calculated using the 2 − ΔΔCt method. A full list of TaqMan assay probes can be found in the Supplemental Table 2. For qPCR measurement of miRNA expression, drug-free naïve *n* = 4, heroin *n* = 8. For measurement of miRNA target mRNA genes, 21D N, *n* = 8; 21D HH, *n* = 12; 21D HL, *n* = 4.

### Western-Blot

10 ug of OFC protein samples (*n* = 8/group) were separated using Western blots as previously described [64]. Membranes were imaged using ImageQuant TL 10.0 analysis software (Cytiva, Marlborough, MD). A full list of antibodies and relative dilutions can be found in Supplemental Table 3.

### Statistical analysis

All data are presented as mean ± standard error of the mean (SEM). Two-way analysis of variance (ANOVA) with repeated measures (RM-ANOVA) were used on self-administration data to compare acquisition behavior over 10 days using the factors of time and lever, and on relapse tests (30 min time-bins) using the factors of treatment and time. Two-way ANOVA was used to examine relapse test results on the sum responses over 90 min, with the factors of treatment and lever, and post hoc comparisons were done using Sidak’s multiple comparisons test. One-way ANOVA was used to compare groups of 3 or more followed by post hoc Tukey tests to control for multiple comparisons. Unpaired two-tailed Student’s t-tests were used to analyze differences between two groups with normal distributions. D’Agostino-Pearson tests were performed to evaluate normality similar levels of variance were observed between groups. A *p* value of less than 0.05 (*p* < 0.05) was considered statistically significant. Log-rank tests were performed on survival curve data to examine differences in the rate of reduced responding during relapse tests. Statistical analyses of qPCR data were performed on ΔΔCT values prior to log transformation of fold change. Small-RNA sequencing, proteomics analysis and infusion of miRNA mimics, inhibitors, or viruses were performed in biological replicate samples from independent cohorts of animals. Results from in vivo infusion of miRNA mimics, inhibitors or viruses after 21D forced abstinence were obtained from 2–3 cohorts of animals per manipulation. qPCR and Western blot validation of small RNA-sequencing and proteomics were performed in biological replicate samples. Sample sizes were determined based on similar studies in this field. No blinding or randomization was performed in this study, due to the necessity to balance heroin intake between treatment groups. Animals were excluded from the analysis if they failed to display a 2-fold preference for the active lever compared to the inactive on the last 3 days of heroin self-administration; failed to make 10 heroin infusions during the last 3 days of heroin self-administration; or were identified as an outlier with a ROUT outlier test. All analyses were performed using the GraphPad software package (Prism version 9; GraphPad, San Diego, California, USA).

## Results

### Incubation of heroin craving regulates OFC miRNA expression

**Fig. 1:**
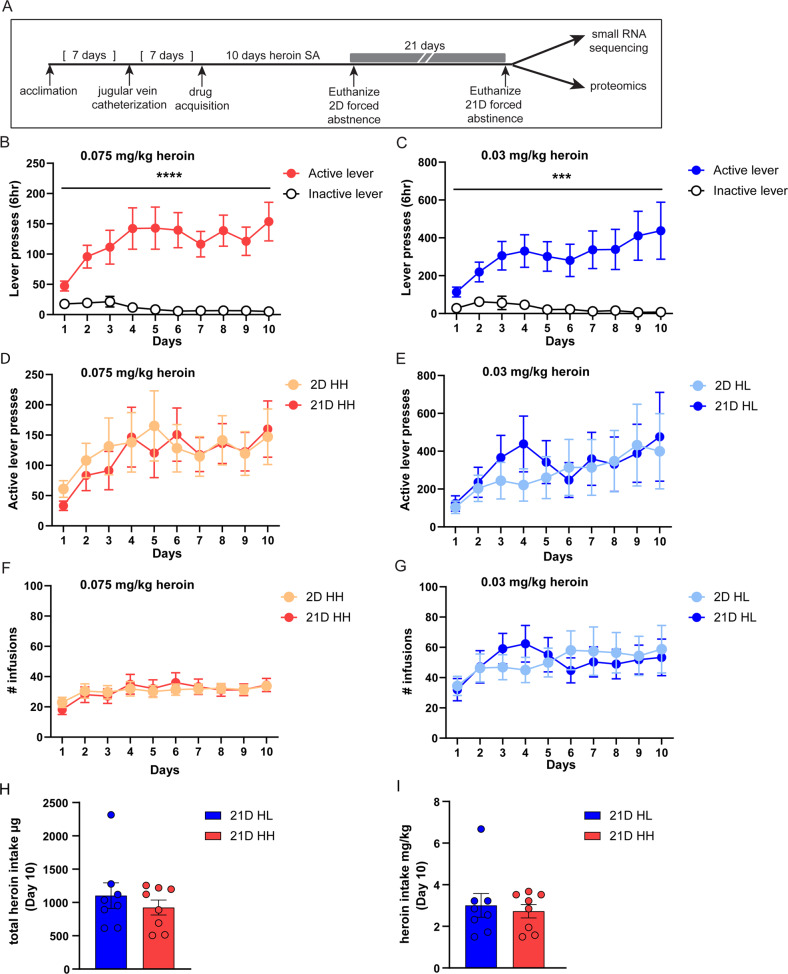
Total heroin intake does not differ for animals that self-administer high or low dosages of heroin in a long-access protocol. **A** Schematic overview of experimental timeline. Rats underwent self-administration (SA) of 0.075 mg/kg/infusion (HH) or 0.03 mg/kg/infusion (HL) for 10 days, followed by forced abstinence in their home cage for 2 or 21D. Animals were then euthanized for molecular analysis of miRNA expression and proteins in the OFC. **B**–**G** Heroin self-administration with 0.075 mg/kg/infusion (left) and 0.03 mg/kg/infusion (right). Displayed are the active vs. inactive lever presses for all animals (**B**, **C**), active vs. inactive lever presses in after-grouped animals (**D**, **F**) and number of infusions (**F**, **G**). **H**, **I** Total heroin intake and heroin relative to body weight at day 10 in HH and HL 21D animals. Error ± S.E.M. ***p < 0.001; ****p < 0.0001.

Our previous data demonstrated that rats display robust incubation of heroin craving behavior after 21 days (D) of forced abstinence [15] from self-administration of 0.075 mg/kg/infusion heroin coupled with discrete drug cues (tone, light). Furthermore, we determined that a lower heroin dosage of 0.03 mg/kg/infusion was not permissive for the incubation of heroin craving. We hypothesized that genes and proteins are differentially regulated between animals that do or do not display incubation behavior, despite similar drug history. To test this hypothesis and elucidate a molecular mechanism that sustains long-lasting heroin-seeking behavior, we first performed a small RNA sequencing analysis to identify small, noncoding miRNAs that are specific for incubation behavior. Animals self-administered either 0.03 mg/kg/infusion (low, HL) or 0.075 mg/kg/infusion (high, HH) heroin in daily 6-hour sessions for 10 days under an FR1 schedule of reinforcement (Fig. 1A). As expected, rats quickly learned to distinguish between the drug reinforcing lever (i.e., active lever) and the inactive lever with both doses (Two-way RM ANOVA, 0.075 mg/kg/infusion time x lever interaction: F(9,414)= 5.399, *p* < 0.0001; main effect of lever: F(1,46)= 23.56, *p* < 0.0001 and Two-way RM ANOVA, 0.03 mg/kg/infusion time x lever interaction: F(9,414)= 3.545, *p* = 0.0003; main effect of lever: F(1,46)= 11.83, *p* < 0.0012; Fig. 1B, C). At the end of the 10 days of self-administration, animals underwent forced abstinence in their home cage for either 2 or 21D. Rats were split into either 2D or 21D abstinence groups with the average number of active lever presses and infusions balanced between the groups (Fig. 1D-1G). We did not observe any significant difference between the two groups for these two parameters (Supplemental Fig. 2A–D). To preclude the notion that molecular changes observed with incubation behavior are simply linked to the amount of heroin administered during the acquisition, we compared the amount of heroin administered by animals at both heroin dosages. HL and HH groups consumed similar amounts of heroin and did not show any statistical difference in total heroin intake (Fig. 1H) or heroin intake in relation to body weight (Fig. 1I) after 10 days of self-administration, suggesting that the amount of heroin consumed is not responsible for molecular changes linked to the incubation behavior. Rather, such incubation-associated molecular changes are likely driven by the dosage of heroin that is coupled with drug cues at the time of infusion, as we have previously theorized [15].

**Fig. 2:**
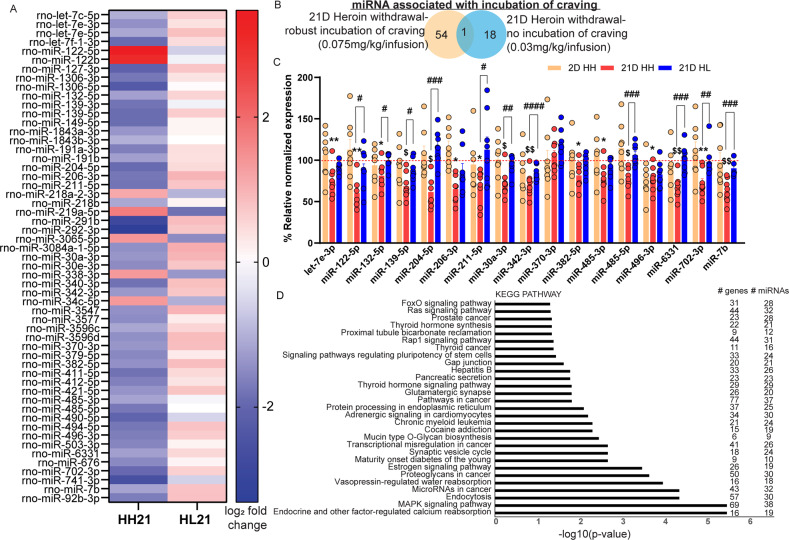
Heroin regulates OFC miRNAs associated with long-term heroin-seeking behavior. **A** Heatmap of differentially expressed miRNAs in 21D HH vs 21D N (left) and 21D HL vs 21D N (right). **B** Venn diagram of miRNAs regulated in 21D HH or 21D HL animals and overlaps between the two groups. **C** qPCR measurement of 17 incubation-associated miRNAs in OFC samples from HH and HL animals. Displayed are the percentage of normalized expression values with respect to drug-naïve animals collected at 2 or 21D time-points. **D** miRPath pathway analysis of putative genes targeted by incubation-related miRNAs. Error ± S.E.M. *p < 0.05; **p < 0.01; ^$^*p* < 0.001; ^$$^*p* < 0.0001, one-way ANOVA. ^#^*p* < 0.05; ^##^*p* < 0.01; ^###^*p* < 0.001; ^####^*p* < 0.0001, Tukey’s multiple comparison test 21D HH vs. 21D HL.

At the end of each abstinence timepoint, rats were euthanized, and the OFC was collected for molecular analysis of miRNAs. Animals were not tested in a relapse test. The aim of this analysis was to identify miRNAs selectively regulated in animals that displayed long-term incubation behavior (i.e., HH 21D animals) and not in those that do not (HL 21D, HH 2D). We chose the OFC because it plays a critical role in the incubation of heroin craving [65–67]. We profiled miRNAs in the OFC of animals that underwent 21D of forced abstinence from both the low (HL) and high (HH) doses of heroin as well as drug-naïve animals that were housed in the animal facility throughout the duration of the experiment. Sequencing analysis detected approximately 720 total known miRNAs in the treatment groups and of these, we found that 55 miRNAs were significantly regulated in the HH group relative to the drug-naïve group (Fig. 2A, Supplemental excel tables SE1,2). 6 miRNAs were significantly upregulated between 21D HH and 21D N, while 49 miRNAs were downregulated. In comparing the expression patterns of the incubation-associated miRNAs in 21D HL animals, we observed nonspecific trends of regulation in the opposite direction: miRNAs upregulated in 21D HH tended to be down in 21D HL and vice versa (Fig. 2A).

To determine which incubation-associated miRNAs may be the most relevant for functional studies, we sought to narrow down the list by identifying the miRNAs selectively regulated in 21D HH animals and not in 21D HL or 2D HH, as well as those that are conserved from rodent to human. The HL group was the ideal control for this study, as these animals endured the same surgical and drug exposure experience as the HH group but do not display incubation of heroin craving. We observed 19 miRNAs significantly regulated in the 21D HL group, of which, 1 was common to both HH and HL groups (Fig. 2B). Thus, 54 miRNAs were associated specifically with incubation of craving in the HH group.

### Validation of miRNAs associated with incubation of heroin craving

Unbiased profiling of small-RNAs has grown in accuracy as the technology has shifted from primarily microarray-based methods to RNA sequencing. However, prior to performing any further study of individual incubation-associated miRNAs in the OFC, we first sought to validate our sequencing findings with quantitative pcr (qPCR). The accuracy of the small-RNA sequencing data was demonstrated by measurement of roughly half of the differentially expressed miRNAs in OFC samples from HH and HL animals that underwent 2 or 21D of forced abstinence compared to their respective controls. We examined the sequence of all putative incubation-associated miRNAs with mirBase and only selected miRNAs that were conserved from rat to human for validation studies. We validated a subset of the sequencing results by performing qPCR to measure levels of 17 out of 54 incubation-associated miRNAs: rno_let-7e-3p; rno_miR-122-5p; rno_miR-132-5p; rno_miR-139-5p; rno_miR-204-5p; rno_miR-206-3p; rno_miR-211-5p; rno_miR-30a-3p; rno_miR-342-3p; rno_miR-370-3p; rno_miR-382-5p; rno_miR-485-3p; rno_miR-485-5p; rno_miR-496-3p; rno_miR-6331; rno_miR-702-3p; and rno_miR-7b (Fig. 2C). We observed significant downregulation of 16 out of 17 miRNAs in the OFC of 21D HH animals relative to their drug-naïve group, but no regulation in 2D HH or 21D HL animals (1 way ANOVA: rno_let-7e-3p, F(2,21) = 7.289, *p* = 0.0039, post hoc HH 21 vs N, *p* = 0.0033; rno_miR-122-5p, F(2,21)=9.110, *p* = 0.0014, post hoc HH 21 vs N *p* = 0.0011, 21D HH vs. 21D HL *p* = 0.0301; rno_miR-132-5p, F(2,21)=5.498, *p* = 0.0120, post hoc HH 21 vs N *p* = 0.0235, 21D HH vs. 21D HL *p* = 0.0241; rno_miR-139-5p, F(2,21)=11.52, *p* = 0.0004, post hoc HH 21 vs N *p* = 0.0003, 21D HH vs. 21D HL *p* = 0.0159; rno_miR-204-5p, F(2,21)=14.64, *p* = 0.0001, post hoc HH 21 vs N *p* = 0.0019, 21D HH vs. 21D HL *p* = 0.0199; rno_miR-206-3p, F(2,21)=4.271, *p* = 0.0278, post hoc HH 21 vs N *p* = 0.0220; rno_miR-211-5p, F(2,21)=5.026, *p* = 0.0165, post hoc HH 21 vs N *p* = 0.0550, 21D HH vs. 21D HL *p* = 0.0199; rno_miR-30a-3p, F(2,21)=10.80, *p* = 0.0006; post hoc HH 21 vs N *p* = 0.0008, 21D HH vs. 21D HL *p* = 0.0045; rno_miR-342-3p, F(2,21)=23.80, *p* < 0.0001, post hoc HH 21 vs N *p* < 0.0001, 21D HH vs. 21D HL *p* < 0.0001; rno_miR-382-5p, F(2,21)=4.878, *p* = 0.0182, post hoc HH 21 vs N *p* = 0.0184; rno_miR-485-3p, F(2,21)=4.718, *p* = 0.0203, post hoc HH 21 vs N *p* = 0.0163; rno_miR-485-5p, F(2,21)=12.99, *p* = 0.0002, post hoc HH 21 vs N *p* = 0.0021, 21D HH vs. 21D HL *p* = 0.0003; rno_miR-496-3p, F(2,21)=5.117, *p* = 0.0155, post hoc HH 21 vs N *p* = 0.0147; rno_miR-6331, F(2,21)=17.15, *p* < 0.0001, post hoc HH 21 vs N *p* = 0.0002, 21D HH vs. 21D HL *p* = 0.0001; rno_miR-702-3p, F(2,21)=9.696, *p* = 0.0010, post hoc HH 21 vs N *p* = 0.0021, 21D HH vs. 21D HL *p* = 0.0038; rno_miR-7b, F(2,20)=20.20, *p* < 0.0001, post hoc HH 21 vs N *p* < 0.0001, 21D HH vs. 21D HL *p* = 0.0005; Fig. 2D). No significant differences were observed for miR-370-3p. All miRNAs showed the same trend of regulation predicted by sequencing analysis with the exception of miR-122-5p, which was predicted to be upregulated in the sequencing analysis. As expected, we did not observe any significant regulation of incubation-associated miRNAs in OFC samples from the 21D HL or the 2D HH groups, suggesting that these miRNAs are specifically linked to long-lasting heroin craving. Using an online software, DIANA-miRPath v3.0 tool, we assessed the regulatory role of the miRNAs we identified as incubation-related, and evaluated whether they shared common pathways [73]. KEGG pathway analysis revealed the most enriched pathways were: “Endocrine and other factor-regulated calcium reabsorption,” “MAPK signaling pathway”,” Endocytosis” and “microRNAs in cancer.” However, the list also included two other pathways containing genes associated with addiction, “cocaine addiction” and “Glutamatergic synapse” (Fig. 2D). To determine the specificity of miRNAs associated with a phenotype of long-lasting heroin craving, we also profiled miRNAs in the OFC of a separate group of animals that underwent sucrose self-administration followed by 21D forced abstinence (SU21D). Three miRNAs were significantly regulated in SU21D animals compared to their naïve counterparts: miR-741-3p, miR-196a-5p and miR-497-5p (Supplemental excel table SE3). Of these changes, only miR-741-3p overlapped with HH21D animals and in the opposite direction, demonstrating that the majority of the incubation-associated miRNAs we detected appear to be specific for heroin.

### miR-485-5p regulates long-lasting heroin-seeking behavior

**Fig. 3:**
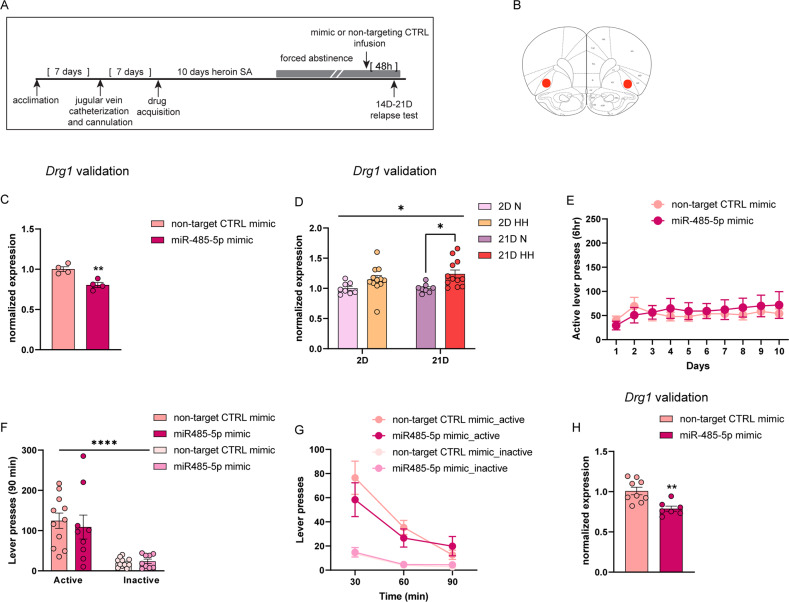
A miR-485-5p overexpression mimic validates regulation of the putative target gene *Drg1* selectively in HH 21D animals but does not regulate heroin craving. **A** Timeline of experimental design. Animals underwent double surgery for OFC cannulation and jugular vein catheter implantation. After 10 days of heroin self-administration (SA), animals underwent forced abstinence for 2-3 weeks. A miR-485-5p mimic or non-targeting CTRL mimic were infused 48 h prior to a relapse test. **B** Representation of the OFC, area of infusion, adapted from Paxinos and Watson’s Brain Atlas. **C** mRNA expression of the predicted miR-485-5p target *Drg1* in the OFC of naïve animals 48 hr after OFC infusion of the miR-485-5p mimic or nontargeting control. **D** *Drg1* mRNA expression in HH animals after 2 or 21D forced abstinence with no relapse test. **E** SA data of animals that subsequently received OFC infusion of a miR-485-5p mimic or nontargeting control mimic. Control mimic, *n* = 9; miR-485-5p mimic. Shown are active lever presses. **F**, **G** Active and inactive lever presses during 90-minute relapse tests 48 h after OFC infusion of the miR-485-5p mimic or nontargeting control from animals that underwent SA followed by 2-3 weeks forced abstinence. **H** *Drg1* expression in animals that underwent functional studies in **E**–**G**. Error ± S.E.M. *p < 0.05; **p < 0.01, ****p < 0.0001.

We observed differential regulation of 54 miRNAs in the OFC of animals that underwent a protocol demonstrated to produce incubation of heroin craving behavior [15], of which we validated 16 with qPCR. We next sought to determine whether the manipulation of an incubation-associated miRNA in the OFC was sufficient to modify long-lasting heroin-seeking behavior after forced abstinence. Among the list of candidates, we selected miR-485-5p, which was downregulated in the OFC after 21D of forced abstinence (Fig. 2C). Regulation of miR-485-5p after 21D forced abstinence was not observed in the nucleus accumbens (Supplemental Fig. 3), another brain region critical for drug reward, indicating that regulation of miR-485-5p after extended abstinence may be, in part, specific to the OFC. miR-485-5p was chosen because it has a known role in synaptic plasticity [74] and we therefore suspected that regulation of miR-485-5p may be a heroin-induced neuroadaptation that contributes to heroin-seeking behavior. Moreover, KEGG pathway analysis identified miR-485-5p as one of the candidate miRNAs involved in common pathways regulating MAPK signaling, cocaine addiction and glutamatergic synapse (Fig. 2D). Craving behavior in the animal model is measured by active lever presses during a relapse test after a period of forced abstinence. Thus, we hypothesized that upregulation of miR-485-5p expression levels could reduce heroin craving behavior, as measured by a reduction in active lever presses during a relapse test. To test this hypothesis, we first infused a synthetic miR-485-5p mimic into the OFC of animals that had previously self-administered heroin, prior to a relapse test after forced abstinence (Fig. 3A, B). To validate this tool, we used an in vivo transfection reagent to deliver the miR-485-5p mimic directly into the OFC of drug-naïve animals and observed a significant increase in miR-485-5p expression within 48 h, as expected (Supplemental Fig. 4A). qPCR measurement of miR-485-5p levels determined that the miR-485-5p mimic increased OFC levels of miR-485-5p approximately 20-fold compared to a nontargeting control mimic (Unpaired t-test t (6) =3.387, *p* = 0.0147; Supplemental Fig. 4A). To further characterize the efficacy of miR-485-5p mimic, we measured the expression of a predicted target of miR-485-5p, *Drg1* (Developmentally Regulated GTP Binding Protein 1) in drug naïve animals injected with miR-485-5p mimic. In general, miRNAs modulate mRNA translation, and therefore, regulate gene expression. Because of this, we expected a downregulation of the target gene *Drg1* when miR-485-5p levels increased. Indeed, *Drg1* was significantly downregulated following infusion of the miR-485-5p synthetic mimic with respect to the nontargeting mimic control group, indicating that *Drg1* is likely a miR-485-5p target gene (Fig. 3C, Unpaired t-test t (6) =4.520, *p* = 0.0040). However, we did not observe any difference in *Drg1* expression in animals that received OFC overexpression of miR-485-5p with scAAV9-EF1a-rno-mir-485-eGFP or miR-485-5p inhibitors (Supplemental Fig. 5A, B). We next measured *Drg1* expression levels in animals that underwent heroin self-administration with forced abstinence to further support our hypothesis that the miR-485-5p pathway is regulated to support long-lasting heroin-seeking phenotypes. Since the levels of miR-485-5p were significantly decreased in the 21D HH group (Fig. 2A, C), we predicted that *Drg1* levels would be increased in HH 21D animals. As predicted, we observed a selective increase in *Drg1* mRNA expression in the OFC of HH 21D but not HH 2D animals (Two-way ANOVA main effect of treatment: F(1,36)=10.52, *p* = 0.0026; no effect of time x treatment interaction; post-hoc 21D N vs. 21D HH, *p* = 0.0430; Fig. 3D). We then examined the effect of the miR-485-5p mimic in our model of incubation of craving. Animals were implanted with bilateral cannulas in the OFC, trained to self-administer heroin for 10 days and then underwent forced abstinence for several weeks. The miR-485-5p mimic or the non-targeting control mimic were infused into the OFC 48 hours (h) before a relapse test. Animals were divided into balanced treatment groups based on their heroin self-administration behavior, with no significant differences observed in heroin self-administration between the treatment groups prior to the relapse test (Fig. 3E). During the relapse test, animals were re-exposed to the self-administration chamber for 90 min and received all visual and auditory cues present during self-administration except no heroin infusions occurred. A two-way ANOVA revealed a significant main effect of lever during the relapse test, indicating that animals responded on the active lever more than the inactive lever, as expected (F(1,18) = 34.42, *p* < 0.0001). No effect of treatment of treatment was observed but animals that received the miR-485-5p mimic tended to make less active lever responses in the first 30 min of the relapse test (Fig. 3F, G). We validated the efficacy of the miR-485-5p mimic transfection in animals that underwent heroin self-administration with forced abstinence and confirmed that the mimic significantly reduced levels of *Drg1* in our experimental manipulation (Unpaired t-test t (14) =3.848, *p* = 0.0018; Fig. 3H).

**Fig. 4:**
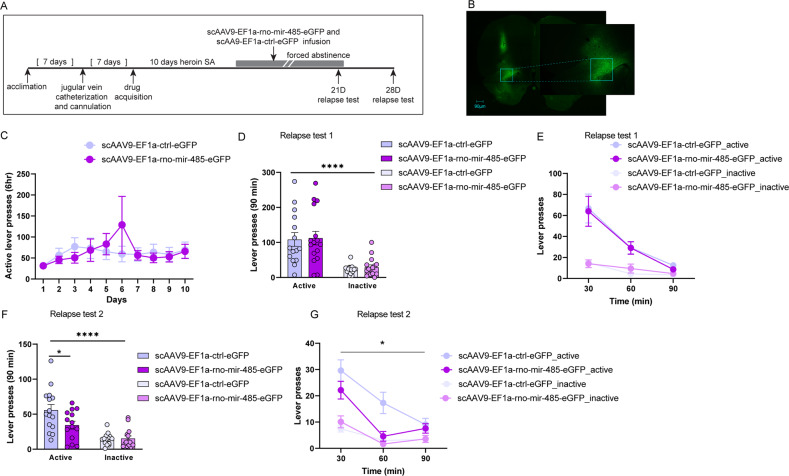
Viral overexpression of OFC miR-485-5p reduces long-lasting heroin-seeking behavior. **A** Overview of experimental timeline. Animals underwent double surgery for OFC cannulation and jugular vein catheter implantation. After 10 days of heroin SA, animals underwent forced abstinence for 3 weeks then were tested for heroin-seeking behavior with two relapse tests, spaced 1 week apart. scAAV9-EF1a-rno-485-eGFP or a scrambled CTRL virus were infused 10-17D prior to the first relapse test. **B** Representative photomicrograph depicting infusion of viral particles into the OFC. Fluorescence indicates GFP reporter signal, 90 µm and magnification of targeted site. **C** SA data of animals that subsequently received OFC infusion of scAAV9-EF1a-rno-485-eGFP or a scrambled CTRL virus. Shown are active lever presses. **D–G** Active and inactive lever presses during 90-minute relapse tests after 21D forced abstinence (relapse test 1, **D**, **E**) and 28D forced abstinence (relapse test 2, **F, G**). Error ± S.E.M. *p < 0.05, ****p < 0.0001.

miRNA mimics are prone to rapid degradation [75, 76] and may not provide a stable elevation of miR-485-5p levels. Additionally, the miR-485-5p mimic resulted in an ~20-fold increase in miR-485-5p levels in the OFC, which represents an extraphysiological environment that may impact our predicted results. Because of this, we next used an alternative viral strategy to overexpress miR-485-5p in the OFC. Infusion of scAAV9-EF1a-rno-mir-485-eGFP into the OFC resulted in a 2-fold increase in miR-485-5p expression relative to infusion of a scAA9-EF1a-ctrl-eGFP control virus (Unpaired t-test t (16) =4.872; *p* = 0.0002; Supplemental Fig. 4B). Using the same experimental timeline as described in Fig. 3, we delivered scAAV9-EF1a-rno-mir-485-eGFP or scAA9-EF1a-ctrl-eGFP into the OFC via cannulas 10-17D before a relapse test in animals that had previously self-administered heroin (Fig. 4A). Rats receiving scAAV9-EF1a-rno-mir-485-eGFP or scAA9-EF1a-ctrl-eGFP were balanced for self-administration behavior (Fig. 4C). Similar to miR-485-5p mimic manipulation, a two-way ANOVA revealed a main effect of lever presses during the entire 90-minute relapse test, as animals made more responses on the active lever than the inactive (F (1,29) = 42.13, *p* < 0.0001; Fig. 4D, E). No main effect of treatment was observed for either lever. However, when animals underwent a second 90-minute relapse test one week later (28 D post self-administration) we observed a significant decrease in active lever presses in animals infused with scAAV9-EF1a-rno-mir-485-eGFP, compared to those that received the scAA9-EF1a-ctrl-eGFP virus, and a significant lever X treatment interaction in addition to a main effect of lever (two-way ANOVA lever X treatment interaction: (F(1,27) = 7.215, *p* = 0.0122; main effect of lever: F(1,27) = 50.39, *p* < 0.0001; post-hoc active lever scAA9-EF1a-ctrl-eGFP vs. active lever scAAV9-EF1a-rno-mir-485-eGFP *p* = 0.0120; Fig. 4F). No significant differences were observed in inactive lever presses between the two treatment groups. A binned time analysis of the relapse test indicated that animals that received the scAAV9-EF1a-rno-mir-485-eGFP virus rapidly extinguished active lever pressing within the first 60 min of the test, (Two-way RM-ANOVA main effect of time: F(1.680, 45.37) = 27.23, *p* < 0.0001; main effect of treatment: F(1, 27) = 4.828, *p* = 0.0368; no interaction; Fig. 4G). This effect we observed following the over-expression of miR-485-5p suggests that miR-485-5p might be important for accelerating the extinction of cue-induced associated behavior linked to long-term heroin seeking. Indeed, a survival curve analysis of performance in the relapse tests indicated that animals that received overexpression of OFC miR-485-5p reduced their active lever presses more rapidly than those that received the scrambled control virus. During the first relapse test, ~43% of animals with the OFC miR-485-5p overexpression virus had reduced their active lever pressing to 25% of their active lever responses on the last day of heroin SA, a common criterion applied to assess extinction of drug-seeking behavior [77, 78], compared to only 20% of animals in the scrambled control group (Supplemental Fig. 6A). A log-rank test of the survival curve of the number of relapse sessions required to reach such criterion was not significant for the first relapse test (Chi-square = 1.203; *p* = 0.273). During relapse test 2, ~93% of animals with OFC miR-485-5p overexpression reached the criterion by the end of the second session, compared to only 53% of scrambled control animals. The log-rank test of the survival curve of relapse test 2 was significantly different between the two groups (Chi-square = 3.954, *p* = 0.047; Supplemental Fig. 6B), indicating that OFC miR-485-5p overexpression during the relapse test was associated with a more rapid reduction in active lever responding on subsequent relapse tests. Immediately after the 2nd relapse test, we euthanized animals and collected the OFC to verify overexpression of miR-485-5p in the targeted area. We observed an ~3-fold increase in miR-485-5p levels in scAAV9-EF1a-rno-mir-485-eGFP animals compared to those with the scAA9-EF1a-ctrl-eGFP virus, confirming that miR-485-5p was elevated throughout the duration of both relapse tests (Unpaired t-test t (14) =3.202, *p* = 0.0064; Supplemental Fig. 4C).

**Fig. 5:**
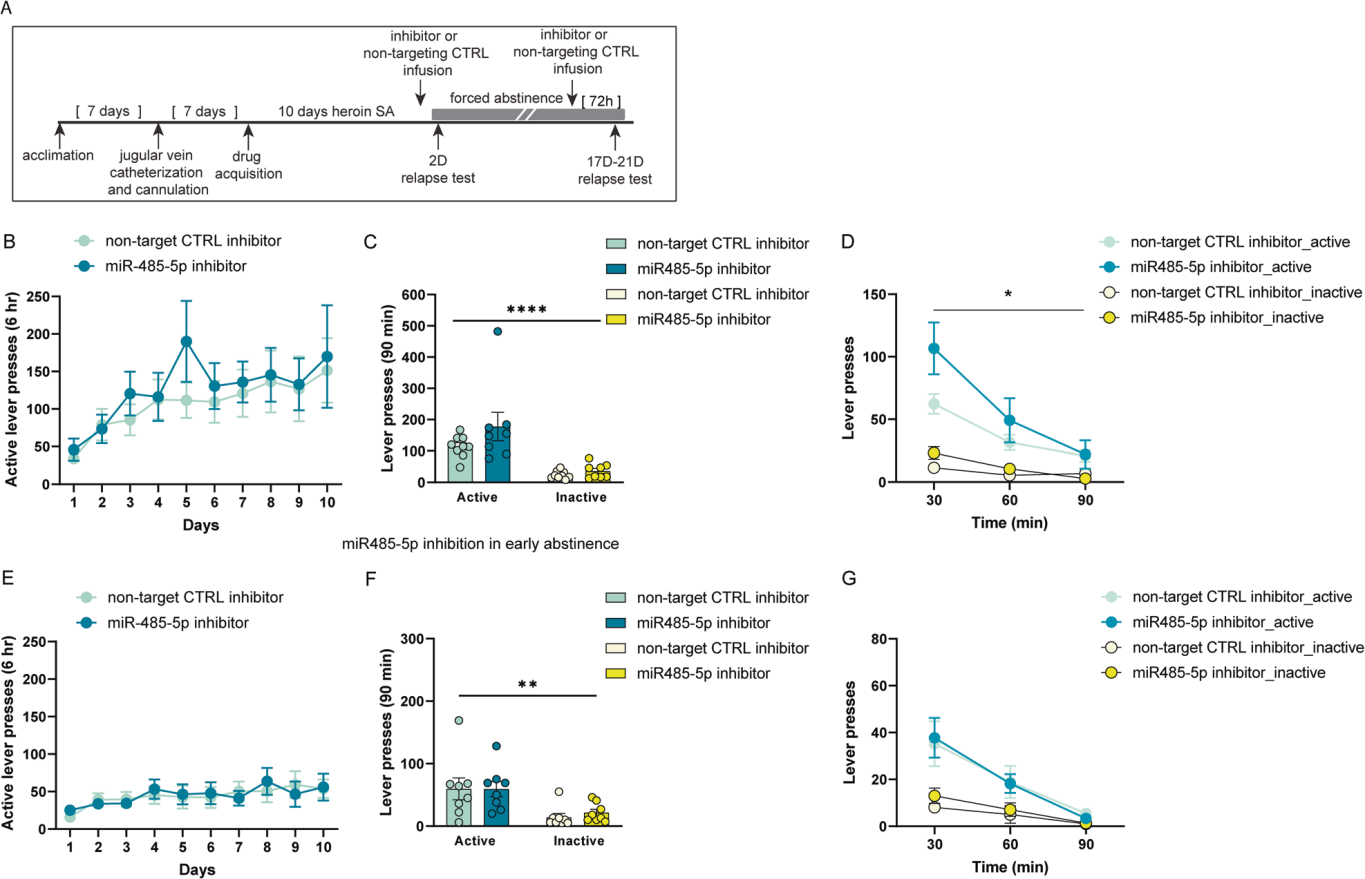
Inhibition of OFC miR-485-5p exacerbates long-lasting heroin-seeking behavior. **A** Timeline of experimental design. Animals underwent double surgery for OFC cannulation and jugular vein catheter implantation. After 10 days of heroin SA, animals underwent acute (2D) or extended (17-21D) forced abstinence. A miR-485-5p inhibitor or non-targeting CTRL inhibitor were infused into the OFC prior to the relapse test. **B–G** Evaluation of miR-485-5p inhibition in late (**B–D**) or early (**E–G**) abstinence. **B**, **E** SA data of animals that subsequently received OFC infusion of a miR-485-5p inhibitor or nontargeting control inhibitor prior to a relapse test in late (**B**) or early (**E**) abstinence. Shown are active lever presses. **C**, **D**, **F**, **G** Active and inactive lever presses during 90-minute relapse tests after OFC infusion of the miR-485-5p inhibitor or nontargeting control inhibitor from animals that underwent 17-21D (**C**, **D**) or 2D abstinence (**F, G**). Error ± S.E.M. *p < 0.05, **p < 0.01, ****p < 0.0001.

The reduction in active lever pressing during the second relapse test when miR-485-5p levels were elevated in the OFC indicates that miR-485-5p regulation contributes to long-lasting heroin-seeking behavior. We sought to provide more evidence to support this hypothesis by inhibiting OFC miR-485-5p expression in our model of incubation of heroin craving to determine if miR-485-5p expression bidirectionally supports long-lasting heroin-seeking behavior. Because miR-485-5p expression was selectively reduced in 21D HH animals that display incubation of heroin craving but not in 2D HH or 21D HL animals that do not display incubation behavior, we hypothesized that low levels of miR-485-5p are associated with increased heroin-seeking behavior. To accomplish this, we used a synthetic miR-485-5p inhibitor that functions as a miRNA sponge and infused it into the OFC. This manipulation resulted in a trend for downregulation of miR-485-5p expression 3 days after the infusion in drug-naïve animals (Unpaired t-test t (5) =2.245, *p* = 0.0750; Supplemental Fig. 4D). Utilizing the same experimental approach as described for the miR-485-5p overexpression experiments, we trained animals to self-administer heroin for 10 days and infused the miR-485-5p inhibitor into the OFC via cannula during forced abstinence, prior to a relapse test (Fig. 5A). Animals in each treatment group (miR-485-5p inhibitor or nontargeting control inhibitor) did not differ in their heroin self-administration behavior prior to forced abstinence (Fig. 5B). Animals underwent forced abstinence for 17-21D, and 72 h before the relapse test were infused with the respective treatment. Heroin-seeking behavior was tested for 90 min with the relapse test. When the seeking behavior was analyzed in the entire 90-minute test, an increase in active lever presses was observed in miR-485-5p inhibitor group, although it did not reach significance (Fig. 5C). A two-way ANOVA of active and inactive lever presses during the 90-minute test revealed a significant main effect of lever, as animals made more responses on the active lever, but no effect of treatment (F(1, 15) = 33.81, *p* < 0.0001; Fig. 5C). However, a two-way RM-ANOVA analysis of active lever responses revealed a significant time X treatment interaction F (2,30) = 3.872, *p* = 0.0319) and main effect of time (F (1.810, 27.16) = 34.21, *p* < 0.0001) throughout the duration of the test (Fig. 5D). Animals that received the miR-485-5p inhibitor pressed the active lever significantly more during the first 30 min of the test compared to those that received the nontargeting control inhibitor (Fig. 5D), supporting the notion that low activity of miR-485-5p is associated with higher levels of heroin seeking behavior. To determine if miR-485-5p selectively regulates long-lasting heroin seeking behavior, we examined the effect of miR-485-5p inhibition on early abstinence. Animals were trained to self-administer heroin for 10 days. Half of the animals received the non-targeting CTRL inhibitor, while the other half received the miR-485-5p inhibitor right after the last SA training session (Fig. 5E). When animals were tested in a 90-minute relapse test after 2D abstinence, both groups showed similar amount of active lever presses in the cumulative 90-min test (Fig. 5F). A two-way ANOVA revealed a significant main effect of lever, as animals preferred the active lever over the inactive (F(1,14) = 16.88, *p* = 0.0011), but no effect of treatment (Fig. 5F). Similarly, no differences were observed between the two groups when examining their behavior patterns in the 30-min time-bin analysis (Fig. 5G). This data suggests that miR-485-5p is more likely linked to the long-term heroin-seeking behavior rather than the early stage of abstinence.

### Identification and validation of miRNA gene targets associated with incubation of heroin craving

In mammalian cells, miRNAs primarily function to regulate gene expression by interacting with their target mRNAs to inhibit protein translation. Thus, to identify miRNA-mediated translational pathways involved in long-lasting heroin seeking, we next evaluated the OFC protein expression associated with incubation of heroin craving by performing label-free proteomics on tissue samples from animals that underwent 21D forced abstinence from heroin self-administration. By overlaying the proteomics data with the small-RNA seq dataset, this approach allowed us to identify both miRNA-dependent and -independent protein networks regulated in animals with long-lasting heroin seeking behavior.

**Fig. 6:**
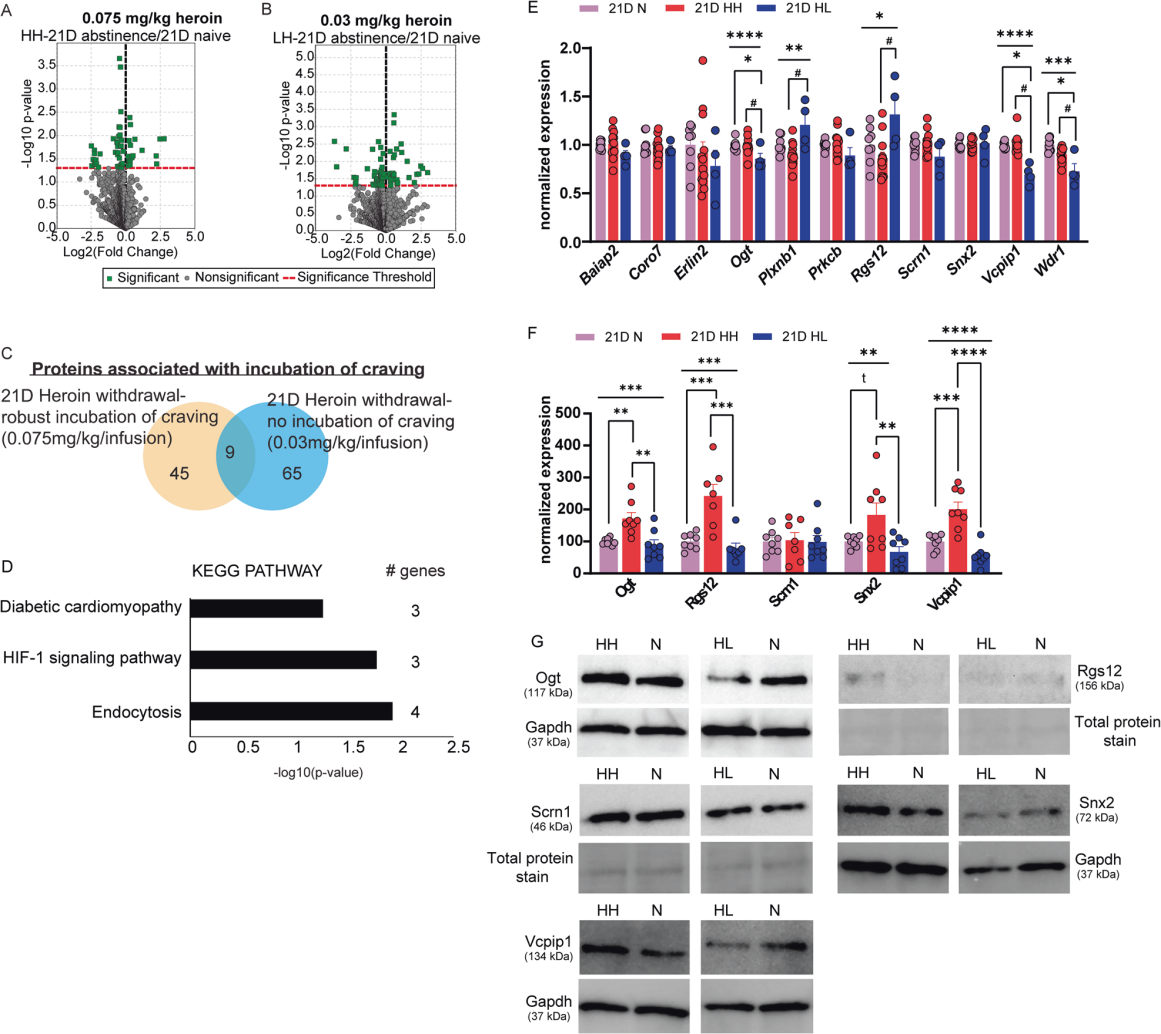
An OFC protein profile associated with long-term heroin-seeking behavior. **A**, **B** Volcano plots of proteins regulated in 21D HH (A) or 21D HL (B) animals, expressed as Log2 fold change. **C** Venn diagram of proteins significantly regulated in the OFC of 21D HH and 21D HL animals. (**D**) DAVID KEGG Pathway analysis of incubation-associated proteins categorized as putative “miRNA-dependent proteins.” **E**, **F** mRNA and protein quantification of incubation-associated genes identified in the proteomic analysis. **E** Shown are relative expression values obtained with qPCR. *p < 0.05, **p < 0.01, ***p < 0.001, ****p < 0.0001; ^#^*p* < 0.05 post hoc test 21D HH vs. 21D HL, *p < 0.05 post hoc test 21D HL vs 21D N. **F** Western Blot quantification of 21D HH targets from the proteomic analysis. *p < 0.05, **p < 0.01, ***p < 0.01 ****p < 0.0001, t = trend. **G** Images of representative treatment group samples from Western Blots with molecular weight of each respective protein. Error ± S.E.M.

The proteomic analysis detected ~3,000 proteins in the OFC and revealed that 54 proteins were significantly regulated in the OFC of the 21D HH group when compared to the 21D naïve group (Fig. 6A). By contrast, 74 proteins were significantly regulated in the 21D HL group relative to naïve (Fig. 6B). 9 proteins overlapped between the two lists, leaving 45 proteins selectively associated with a phenotype of incubation of heroin craving (Fig. 6C). We then examined the overlap of the small-RNA seq data and the proteomics data to determine whether any incubation-associated miRNAs targeted the differentially regulated OFC proteins identified by the proteomics analysis. Using miRNA target prediction, we determined that 37 incubation-associated proteins were predicted to be targeted by the incubation-associated miRNAs (Supplemental Table 4). Therefore, we termed this list ‘miRNA-dependent proteins’ associated with persistent heroin seeking. 7 miRNAs (rno-let-7e-3p, rno-miR-122b, rno-miR-1306-3p, rno-miR-3084a-1-5p, rno-miR-340-3p, rno-miR-412-5p and rno-miR-676) are not present in Supplemental Table 4 because they were not predicted to target any of the proteins we detected in the proteomic analysis.

Similar to the pathway analysis of miRNAs linked to incubation, we used DAVID to perform a KEGG pathway functional enrichment analysis on proteins associated with long-lasting heroin seeking in HH animals [70]. KEGG pathway analysis revealed only three enriched pathways involving the list of predicted miRNAs-regulated proteins that belonged to the following terms: “Diabetic cardiomyopathy,” “HIF-1 signaling pathway” and “Endocytosis” (Fig. 6D). Interestingly, both the miRNA pathway analysis and the protein pathway analysis commonly identified the endocytosis pathway as a significantly enriched pathway among the regulated protein list (Fig. 2D, Fig. 6D).

To further explore miRNA-mediated and -independent protein pathways associated with incubation of heroin craving, we examined mRNA and protein expression of putative incubation-associated proteins in the OFC of animals that self-administered heroin. We selected our candidates based on previously reported functions linked with drug addiction, synaptic plasticity, and memory. Using qPCR, we first measured mRNA expression of 11 genes regulated in HH animals in the proteomics analysis. One-way ANOVA revealed significant regulation of *Ogt*, *Plxnb1*, *Rgs12*, *Vcpip1* and *Wdr1* (One-way ANOVA *Ogt:* F(2, 18) = 78.33, *p* < 0.0001, post hoc 21D HH vs 21D HL *p* < 0.0001, 21D HL vs 21D N *p* < 0.0001; *Plxnb1*: F(2, 21) = 6.995, *p* = 0.0047, post hoc 21D HH vs 21D HL *p* = 0.0037; *Rgs12*: F(2, 21) = 5.402, *p* = 0.0128, post hoc 21D HH vs 21D HL *p* = 0.0104; *Vcpip1*: F(2, 21) = 28.42, *p* < 0.0001, post hoc 21D HH vs 21D HL *p* < 0.0001, 21D HL vs 21D N *p* < 0.0001; *Wdr1*: F(2, 21) = 12.79, *p* = 0.0002, post hoc 21D HH vs 21D HL *p* < 0.0037, 21D HL vs 21D N *p* = 0.0001, Fig. 6E).

Lastly, because mRNA expression changes may be compensatory to regulated protein levels, we next sought to validate the proteomics data by quantifying protein expression levels of predicted incubation-associated proteins in 21D HH, HL and drug-naïve animals using Western Blot analysis (Fig. 6F). Although we attempted to measure the expression of 10 proteins in our samples, we were only able to measure 5 proteins due to poor antibody quality: Ogt, Rgs12, Scrn1, Snx2 and Vcpip1. Ogt and Rgs12 were predicted targets of miR-485-5p, while the other three proteins are predicted targets of multiple incubation-associated miRNAs. We found that Ogt, Rgs12, Snx2 and Vcpip1 were significantly elevated in HH animals, compared to both HL and naïve groups, suggesting these proteins might be involved in the incubation of craving behavior (One-way ANOVA Ogt: F(2, 21) = 10.12, *p* = 0.0008, post hoc 21D HH vs. 21D N *p* = 0.0049, 21D HH vs 21D HL *p* = 0.0014; Rgs12: F(2, 19) = 15.54, *p* = 0.0001, post hoc 21D HH vs. 21D N *p* = 0.0006, 21D HH vs 21D HL *p* = 0.0002; Snx2: F(2, 21) = 6.373, *p* = 0.0069, post hoc 21D HH vs. 21D N *p* = 0.0626, 21D HH vs 21D HL *p* = 0.0070; Vcpip1: F(2, 21) = 24.32, *p* < 0.0001, post hoc 21D HH vs. 21D N *p* = 0.0003, 21D HH vs 21D HL *p* < 0.0001). Since *Ogt* and *Rgs12* were predicted to be miR-485-5p targets, we analyzed how the expression of these targets may change in animals that received overexpression of OFC miR-485-5p with either the miR-485-5p mimic and AAV, or inhibition with the miR-485-5p inhibitor. In animals that received the miR-485-5p overexpression virus in the OFC, a two-way ANOVA revealed a significant time X treatment interaction for *Ogt* (F(1,14) = 8.546, *p* = 0.0111; post hoc: scAAV9-EF1a-ctrl-eGFP vs. scAAV9-EF1a-rno-mir-485-eGFP 15D *p* = 0.0021; Supplemental Fig. 5D) and main effect of treatment for *Rgs12* (F(1,14) = 4.654, *P* = 0.0489; Supplemental Fig. 5G). No differences in *Ogt* or *Rgs12* expression were observed in the OFC of naïve animals that received the miR-485-5p mimic or inhibitor when compared to their respective control group (Supplemental Fig. 5C, E, F, H). These data reveal that both *Ogt* and *Rgs12* are partially responsive to the manipulation of miR-485-5p levels.

## Discussion

This study is the first report of miRNA regulation in the OFC following opioid exposure. Maintenance of long-lasting opioid-seeking behavior is supported by the OFC [65–67]. In studies with human heroin-dependent patients, the OFC shows activation when patients are presented with drug-associated cues [68, 79]. Indeed, preclinical models have demonstrated that cells are activated in the OFC upon re-exposure to drug-paired cues following a period of forced abstinence [65]. Previous studies have investigated altered microRNAs in response to stimulants such as cocaine, methamphetamine and nicotine and depressant-like drugs such as alcohol in the nucleus accumbens (NAc) [33–35, 37, 38, 42, 44], dorsal striatum [43], and medial prefrontal cortex [45]. Similarly, miRNAs were found to be altered following opioids in the NAc [60, 62, 80], hippocampus [57] and dorsal ganglia [54]. Our study is the first to examine miRNA regulation in the OFC for any class of drugs and we report a novel miRNA profile unlike those observed in other brain regions for opioid exposure.

We identified a pattern of 54 miRNAs that are regulated in the OFC following 21D of forced abstinence from heroin self-administration. Among these, some have been previously identified as differentially expressed in response to cocaine, alcohol, methamphetamine, as well as opioids. For example, let-7 family, miR-127-3p and miR-30 were found to be overexpressed in the NAc of chronic cocaine-treated rats by sequencing analysis [81]. We found all of them to be downregulated in the OFC following 3 weeks of forced abstinence. On the contrary, miR-370 was the only one that was found changed in the same direction as our analysis [81]. miR-132 was extensively studied in response to cocaine self-administration. It was found to be upregulated in the dorsal striatum [39] but down-regulated in the NAc core after cocaine [42]. miR-132 upregulation was persistent until 10 days after extinction [41] and up-regulated following extinction and reinstatement [42]. Related to cocaine and extinction, miR-191 and miR-382 were up-regulated in the hippocampus of rats in the extinction phase of the conditioned place preference (CPP) [82]. Interestingly, miR-206 was found to be increased in the serum of heroin users, while we observed a significant downregulation of this miRNA in our study [58]. miR-206 was also upregulated in the mPFC in rats after alcohol self-administration [45]. We observed a general decrease in miRNA expression in the OFC of HH animals, with only a few miRNA candidates upregulated, which included miR-218 and miR-219. However, miR-218 and miR-219 were downregulated in the NAc after chronic heroin exposure and in the dorsal root ganglia after chronic morphine treatment, in rodent models [54, 55]. Differences between our findings and those reported in the literature may be due to brain-region-specific miRNA expression profiles, behavioral protocols, and the timing of tissue collection (e.g.- acute versus extended abstinence). We expect that each brain region may elicit a unique miRNA profile that is drug-specific and even behavior-specific. Finally, miR-485-5p was downregulated in methamphetamine-treated rats in striatal regions [49], which is in line with our findings. We did not detect regulation of miR-485-5p in the NAc of HH 21D animals (Supplemental Fig. 3) and therefore conclude that heroin-induced regulation of miR-485-5p after extended forced abstinence has some specificity for the OFC. However, we cannot exclude that the changes we observed might be beyond the OFC area for the other miRNAs we have identified as associated with long-lasting heroin seeking.

A limitation of the current study is that all experiments were conducted solely in male animals. Because of this, we are unable to conclude that the mechanisms described in our study are generalized to both sexes or specific to only male animals. This limitation may also extend to the translatability of our findings, as documented differences in opioid craving behavior between human male and female suggests that sex-specific mechanisms exist to support opioid craving [83]. Nevertheless, very few other studies have explored miRNA regulation associated with long-lasting drug-seeking behavior. Two studies have reported alteration of miRNAs in response to cocaine reinstatement following extinction [37, 42]. In the opioid field, three studies have characterized the effects of miRNAs following forced abstinence from drug exposure. However, the incubation model used in those papers differs from what has been used in the present study. Two of the studies used a conditioned place preference paradigm to test preference following forced abstinence from experimenter-administered opioids [60, 62]. Other studies have demonstrated that miRNA signaling in the NAc can impact opioid self-administration behavior. Overexpression of miR-181 in the NAc of heroin SA rats decreased heroin seeking after 14D of forced abstinence [61]. Similarly, NAc miR-218 inhibited heroin-induced reinforcement in both conditioned placed preference and self-administration [55, 61]. In contrast, overexpression of miR-9 in the NAc increased self-administration of the opioid oxycodone, resulting in burst episodes of intake and a reduction in the interval between infusions [84]. At present, our study is the first and only one that has studied miRNA alteration in the OFC in a self-administration incubation model.

We focused our study on miR-485-5p, which was significantly downregulated after 21D of forced abstinence. miR-485-5p has previously been shown to be downregulated in the striatum in response to methamphetamine [49] and participates in synaptic plasticity events [74]. Such events have been demonstrated to be critical for heroin-induced chronic drug seeking [85]. Interestingly, miR-485-5p was predicted to be involved in cocaine addiction and glutamate synapses in the miRPath pathway analysis. Reducing miR-485-5p levels with a sponge-like inhibitor prior to a relapse test resulted in an exacerbation of heroin seeking at the onset of the relapse test. Overexpression of miR-485-5p did not impact heroin craving at the first relapse test. However, it steadily reduced the motivation for heroin-seeking behavior at the second relapse test, as reflected by a significant reduction in lever presses, suggesting its role as pro-extinction factor or modulator of reconsolidation of heroin-associated memories. Thus, the elevation of miR-485-5p levels during re-exposure to the heroin-paired cues may accelerate and facilitate the extinction of heroin seeking. Conversely, we conclude that the reduction of OFC miR-485-5p levels does the opposite and functions to maintain long-lasting heroin seeking. The miR-485-5p-mediated mechanisms contributing to such behavior still remain unknown. We hypothesized that miR-485-5p animals are able to destabilize the cue-induced learned memory acquired during self-administration at the first relapse test, and thus, easily adapt to the new situation when re-presented for the second time. Glutamate has been shown to be important in the reinstatement process, as, blockage of the glutamate receptor has been shown to facilitate or block reinstatement [86]. Rgs12, a putative miR-485-5p target significantly regulated in our incubation model, was previously reported to be linked to the glutamate signal pathway [87]. Ogt, another miR-485-5p target, has previously been shown to be linked to memory and cognitive function [88, 89]. Knocking down Ogt impaired learning and memory in mice and low levels of Ogt are associated with synaptic plasticity and cognitive impairments. [88, 89]. In our study, Ogt was upregulated in HH animals that typically have persistent heroin-seeking behavior, suggesting that the cognitive-linked function of Ogt may support continued heroin-associated memory to delay extinction of heroin-seeking during the relapse test. Because of this, we hypothesize that overexpression of miR-485-5p presumably downregulates Ogt protein and we can therefore speculate that the low level of Ogt ultimately impairs the cue and context associated heroin memory previously established. The end result is the rapid promotion of the extinction of heroin seeking in the miR-485-5p overexpression animals. miR-485-5p may act through Ogt (Supplemental Fig. 5C–E) or Rgs12 (Supplemental Fig. 5F–H) by interfering with the glutamate signaling (Rgs12) or impairing cognition and synaptic plasticity (Ogt) to destabilize cue-induced heroin memory and allow for re-adaptation to a new, non-drug environment.

Moreover, other miR-485-5p targets which we were not able to validate with Western blots have been shown to be linked to memory. For example, Wdr1 has been reported to play a predominant role in regulating synaptic plasticity and memory as shown in a study employing Wdr1 knockout mice [90]; and Baiap2 is a modulator of human memory strength [91]. Therefore, we cannot exclude the involvement of these two targets as well in the pro-extinction hypothesis of miR-485-5p. It should be noted that although we validated Drg1 as a target of mir-485-5p in our functional studies, Drg1 was not detected in any OFC proteomics samples. miR-485-5p manipulation in early abstinence did not induce any alteration as observed using the miR-485-5p inhibitor. Thus, we hypothesize that miR-485-5p is more linked to long-term heroin seeking rather than the early abstinence period. In conclusion, we identified a novel miRNA profile significantly altered in the OFC in response to the incubation of heroin craving. We demonstrated OFC miR-485-5p and its putative target genes are associated with long-term heroin seeking and manipulation of OFC miR-485-5p bidirectionally alters long-term seeking behavior.

### Supplementary information

Supplemental material

Supplemental excel tables
