# Supplementary material for: Orbitofrontal cortex microRNAs support long-lasting heroin seeking behavior in male rats
Source: Transl Psychiatry. 2023 Apr 8;13:117. doi: 10.1038/s41398-023-02423-4 (PMC10082780; doi:10.1038/s41398-023-02423-4)
Supplement: Supplementary file 1 — Supplemental material [file 41398_2023_2423_MOESM1_ESM.docx]

Supplemental materials for “Orbitofrontal cortex microRNAs support long-lasting heroin seeking behavior in male rats” by Zanda et al:

Supplemental experimental methods:

**Animal surgeries**

For drug self-administration, rats were deeply anesthetized with isoflurane (3% induction, 2-2.5% maintenance) and were implanted with silastic tubing catheters (SAI Infusion Technologies, Lake Villa, IL, USA) in the right jugular vein under aseptic conditions as previously described [1]. Catheters were flushed with heparinized saline solution every day during recovery and self-administration sessions. For direct injection of synthetic miRNA mimics, inhibitors or viruses in animals that did not undergo self-administration, compounds were infused directly in the OFC using 10 µl Hamilton syringes attached to a microinjection system pump (World Precision Instrument, Sarasota, Florida, USA) over 5 minutes. Bilateral injections of 1ul/hemisphere were made into the OFC using the following stereotaxic coordinates (from Bregma): anteroposterior (AP) +3.2 mm; mediolateral (ML) ±3.3 mm (10°angle); dorsoventral (DV) -5.1 mm. The syringe was kept in place for 5 minutes following infusion. For delivery of synthetic miRNA mimics, inhibitors or viruses in animals following heroin self-administration, rats were implanted with permanent bilateral guide cannulas (23-gauge, Plastic One, Roanoke, Vermont, USA) 1.0 mm above OFC, immediately following the catheterization procedure. The stereotaxic coordinates from Bregma were anteroposterior (AP) +3.2 mm; mediolateral (ML) ±3.3 mm (10°angle); dorsoventral (DV) -4.1 mm as previously described [2]. Following surgery, animals were singly housed for the remainder of the study to prevent damage to their catheters and/or cannula. During the post-surgical recovery period, rats were monitored daily for weight and behavior changes.

**Self-administration procedure, relapse test and OFC infusion**

Drug self-administration studies was performed as previously described (Zanda et al., 2021). Active lever pressing resulted in the activation of the infusion pump for intravenous infusion of 0.075mg/kg/infusion (0.1ml infusion volume over 5.64 seconds, pump rate 0.01773 ml/s) of heroin solution, presentation of a 65db, 2.9kHz acoustic cue and illumination of a stimulus light above the active lever, followed by a 20-second time out period where the lever was not responsive. Before starting self-administration, catheter patency was tested with 1% propofol and animals were food restricted to take 10% of their body weight. Food-restriction during self-administration did not decrease bodyweight and was used to increase motivation to perform the task (Supplemental Figure 1). A total volume of 1ul of miRNA mimics, inhibitors or viruses were infused into each hemisphere of the OFC and the injectors were kept in place for an additional 5 minutes. Mimics were delivered 48 hours prior to a relapse test; hairpin inhibitors were delivered 72 hours before relapse tests; and viruses were delivered 10-17 days before relapse test 1. During relapse tests, animals were placed back into the SA chamber for three consecutive 30 minute relapse tests that totaled 90 minutes. Animals received exposure to all auditory and visual cues that were present during SA, except active lever pressing did not result in any infusions. Animals infused with the mir-485-5p mimic were tested for relapse after 14 or 21 days abstinence, while those infused with the mir-485-5p inhibitor were tested for relapse after 17 or 21 days abstinence, due to COVID-19 restriction. Cannula placement was examined at the conclusion of the study. Sucrose self-administration was performed in uncatheterized animals for 10 days in 2-hour daily sessions under an FR1 schedule of reinforcement. Pressing the active lever resulted in delivery of a single chocolate-flavored sucrose pellet (45 mg, Bio-Serv, Flemington, NJ, USA). At the end of self-administration training, animals underwent 21D of abstinence in their homecage. Sucrose animals were euthanized after abstinence without a relapse test.

**Tissue collection and protein extraction**

For molecular profiling of miRNA and protein expression in animals that underwent SA, rats were split into two groups, and were balanced based on the average of active lever presses and infusions. Animals underwent forced abstinence for either 2 or 21D in their home cage. At the end of the abstinence time period, animals were euthanized with decapitation under anesthesia (3% isoflurane). Brains were removed and put in ice-cold isopentane for flash freezing and subsequently transferred on dry ice. Brains were kept at -80°C until dissection. Brains were dissected on dry ice using a temperature-controlled block to maintain RNA integrity. OFC was isolated and removed by punching the area with a sample corer (1.0 mm, Fine Science Tool). Following cell lysis, protein samples were sonicated and centrifuged at 4°C at 12,500 RPM for 10 minutes to remove cell debris. The supernatant was analyzed for yield with the Thermo Scientific bicinchoninic acid (BCA) Assay (Thermo Scientific) and then stored at -80°C until use.

**miRNA library preparation, sequencing, and data analysis**

Total RNA was assessed for quality before library preparation and RNA Integrity Numbers (RIN) were ≥ 7.5 with 28S/18S ≥ 1.3. Small RNAs (18-30 nucleotides) were separated from total RNA by PAGE gel. Selected small RNAs were ligated with 3’ and 5’ adapters. The formed strands were reverse transcribed to cDNA and PCR amplified with high-ping polymerase. The PCR products (100-120bp) were separated by PAGE gel to eliminate primer-dimers and other byproducts then purified. The PCR yield was quantified followed by single strand DNA cyclization (ssDNA circle) for final library construction. DNA nanoballs (DNBs) were generated by ssDNA circle by rolling circle replication. The DNBs were then loaded onto the BGISEQ-500 platform and sequencing of 100bp paired-end reads was performed. Raw sequencing reads were filtered to remove reads with low quality tags, 5’ primer contaminants, tags without a 3’ primer, tags with poly A and those shorter than 18nt. Bowtie2 was used to align clean reads to the reference genome [4]. miRNA expression levels were calculated as Transcripts Per Kilobase Million (TPM) to directly compare differences in gene expression between samples.

**Proteomic analysis**

A chloroform-methanol precipitation was carried out to dehydrate protein, followed by dual enzymatic digestion with Lysine and Trypsin. Samples were acidified/quenched with trifluoroacetic acid (20% TFA). Salt was removed with MacroSpin columns. The effluents were dried and re-suspended in formic acid (70% FA) and TFA (0.1%) to obtain the total digestion product. Label-Free Quantification (LFQ) was operated on Orbitrap Fusion mass spectrometer (ThermoFisher Scientific) connected to a Waters nanoACQUITY UPLC system equipped with a Waters Symmetry® C18 180 μm × 20 mm trap column and a 1.7-μm, 75 μm × 250 mm nanoACQUITY UPLC column (35°C). The total digestion product was diluted in 0.1% TFA, and samples were injected in duplicate. A resolution of 120,000 and 60,000 was utilized for MS and MS/MS scans, respectively, to ensure high quality read. High-energy Collisional Dissociation (HCD) MS/MS spectra filtered by dynamic exclusion was acquired over a 3 second duty cycle for charge states 2-8 with m/z isolation window of 1.6. 97% Buffer containing 0.1% FA in water and 3% Buffer containing 0.075% in acetonitrile (ACN) were used for the trapping step, followed by linear gradient elution with the FA/CAN buffer.

**miRNA and mRNA qPCR**

Each sample was run in triple biological replicate using the miRCURY LNA SYBR Green PCR Kit and locked nucleic acid (LNA) SYBR green primers from Qiagen, as previously described [8]. RNU5G and rno-miR-320-3p were used as endogenous control genes. A full list of miRNAs primers can be found in Supplemental Table 1. For mRNA qPCR, 50 ng of total RNA was reverse transcribed into cDNA using qScript XTL cDNA SuperMIX (Quantbio, Beverly, MA) in a MiniAmp Thermal Cycler (Thermo Fisher Scientific) and qPCR was performed with Perfecta Fastmix II (Quantbio, Beverly, MA) and Taqman assays probe in a Quantstudio 3 thermal cycler, as previously described [9]. Beta-actin (Actb) and glyceraldehyde 3-phosphate dehydrogenase (GAPDH) were used as endogenous control genes for mRNA normalization.

**Western-Blot**

Protein samples were separated using Midi-Protean TGX Gels (Bio-Rad Laboratories, Hercules, California) in a Midi ProTEAN Tetra Cell (Bio-Rad Laboratories) at 250V for 20 minutes. Proteins were then transferred to a polyvinylidene fluoride membrane (PVDF) (Immobilon- FL Membrane, Merck Millipore, Burlington, MA) at 100V for 30 min [3]. Membranes were rinsed in water then methanol and completely dried. Nonspecific sites were blocked with 5% Dry Milk Powder (Dry Milk Powder, Research Products International, Mt Prospect, IL) diluted in phosphate-buffered saline containing 1% Tween-20 (PBST) (Fisher Bioreagents, Fair Lawn, NJ) for 1 hour. Membranes were incubated overnight on a shaker at 4 °C with the primary antibodies diluted in 5% milk/PBST blocking solution. Membranes were washed 4 x 5 minutes in PBST on a shaker at room temperature to remove unbound antibody. Membranes were then incubated with HRP-conjugated secondary antibody (Anti-Rabbit IgG HRP Conjugate, Promega, Madison, WI) at a 1:10,000 dilution in blocking solution for 2 hours on a shaker at room temperature. Excess antibody was removed by washing membranes 3 x 5 minutes in PBST and once in PBS on a shaker at room temperature. Membranes were incubated in SuperSignal West Pico PLUS Chemiluminescent Substrate (Thermo Scientific) for 5 minutes at room temperature on a shaker and then imaged. Proteins were normalized to GAPDH, when possible, or to the total protein stain when the molecular weight of a protein of interest was too close to the GAPDH band.

**Immunohistochemistry**

Animals were deeply anaesthetized with ketamine and perfused with 1X phosphate buffered saline solution (PBS) followed by 4% paraformaldehyde (PFA) solution. The brain was taken out from the skull and post-fixed overnight at 4° C in 4% PFA, and subsequently stored in 30% sucrose/PBS solution. Brains were washed in PBS, dried with tissue paper, embedded in Optimal Cutting Temperature (OCT) reagent and frozen at -20° C inside a cryostat. Brains were cut at 50 µm thick sections. Floating tissue sections were washed in 6-wells plates with 1X PBS. Sections were washed 3 times in tris-buffered saline with 0.25% Triton x-100 (TBS-T) for tissue permeabilization, and then blocked in 5% Normal Goat serum in TBS-T (blocking buffer) for 1 hour. Sections were incubated with Green Fluorescent Protein (GFP) primary antibody (Rabbit monoclonal, ThermoFisher scientific), diluted 1:200 in blocking buffer at 4° C overnight. Sections were washed 3 times, 5 minutes/each with TBS-T and incubated with Alexa Fluor 488-conjugated Donkey Anti-Rabbit (1:1000, ThermoFisher scientific) for 2 hours at room temperature. Following sequential washes, 3 times in TBS-T and once in TBS, sections were mounted on slides with one drop of mounting media (Prolong Diamond Antifade mountant with DAPI, Invitrogen, ThermoFisher scientific) and covered by coverslips. Slides were dried and stored at -20 ° C. Images were acquired with a NIKON A1R+ Resonant Scanning confocal microscopy imaging system.

**Supplemental Table 1: List of miRNA primers used for qPCR validation:**

| **PCR Assay name** | **Sequence** | **Catalog #** |
| --- | --- | --- |
| hsa-let-7e-3p miRCURY LNA miRNA PCR Assay | **MIMAT0025070: 5'CUUUGGUGGCUUAGUUCUUUGUGC** | YP00205301 |
| hsa-miR-122-5p miRCURY LNA miRNA PCR Assay | **MIMAT0000421: 5'UGGAGUGUGACAAUGGUGUUUG** | YP00205664 |
| hsa-miR-132-5p miRCURY LNA miRNA PCR Assay | **MIMAT0004594: 5'ACCGUGGCUUUCGAUUGUUACU** | YP00204552 |
| mmu-miR-139-5p miRCURY LNA miRNA PCR Assay | **MIMAT0000656: 5'UCUACAGUGCACGUGUCUCCAG** | YP00204037 |
| hsa-miR-204-5p miRCURY LNA miRNA PCR Assay | **MIMAT0000265: 5'UUCCCUUUGUCAUCCUAUGCCU** | YP00206072 |
| hsa-miR-206 miRCURY LNA miRNA PCR Assay | **MIMAT0000462: 5'UGGAAUGUAAGGAAGUGUGUGG** | YP00206073 |
| mmu-miR-211-5p miRCURY LNA miRNA PCR Assay | **MIMAT0000668: 5'UUCCCUUUGUCAUCCUUUGCCU** | YP00205091 |
| hsa-miR-30a-3p miRCURY LNA miRNA PCR Assay | **MIMAT0000088: 5'CUUUCAGUCGGAUGUUUGCAGC** | YP00204457 |
| hsa-miR-342-3p miRCURY LNA miRNA PCR Assay | **MIMAT0000753: 5'UCUCACACAGAAAUCGCACCCGU** | YP00205625 |
| hsa-miR-370-3p miRCURY LNA miRNA PCR Assay | **MIMAT0000722: 5'GCCUGCUGGGGUGGAACCUGGU** | YP00204011 |
| hsa-miR-382-5p miRCURY LNA miRNA PCR Assay | **MIMAT0000737: 5'GAAGUUGUUCGUGGUGGAUUCG** | YP00204169 |
| rno-miR-485-3p miRCURY LNA miRNA PCR Assay | **MIMAT0017222: 5'CAUACACGGCUCUCCUCUCUUC** | YP02116189 |
| hsa-miR-485-5p miRCURY LNA miRNA PCR Assay | **MIMAT0017222: 5'CAUACACGGCUCUCCUCUCUUC** | YP02112548 |
| rno-miR-496-3p miRCURY LNA miRNA PCR Assay | **MIMAT0012860: 5'AGUAUUACAUGGCCAAUCUCC** | YP02100800 |
| rno-miR-6331 miRCURY LNA miRNA PCR Assay | **MIMAT0025070: 5'CUUUGGUGGCUUAGUUCUUUGUGC** | YP02108144 |
| rno-miR-702-3p miRCURY LNA miRNA PCR Assay | **MIMAT0017885: 5'UGCCCACCCUUUACCCCACUCCA** | YP02110687 |
| mmu-miR-7b-5p miRCURY LNA miRNA PCR Assay | **MIMAT0000088: 5'CUUUCAGUCGGAUGUUUGCAGC** | YP00204457 |
| hsa-miR-320a miRCURY LNA miRNA PCR Assay | **MIMAT0000678: 5'UGGAAGACUUGUGAUUUUGUUGU** | YP00205082 |
| RNU5G miRCURY LNA miRNA PCR Assay |  | YP00203908 |

**Supplemental Table 2: List of mRNA primers used for gene expression analysis:**

| **Probe Gene Assay name** | **Dye** | **Catalogue number** |
| --- | --- | --- |
| *Baiap2* Rn00589411_m1 | **FAM-MGS** | 4331182 |
| *Coro-7* Rn01424788_m1 | **FAM-MGS** | 4331182 |
| *Erlin2* Rn01482546_m1 | **FAM-MGS** | 4351372 |
| *Ogt* Rn00820779_m1 | **FAM-MGS** | 4331182 |
| *Plxnb1* Rn01746615_m1 | **FAM-MGS** | 4351372 |
| *Prkcb* Rn00562312_m1 | **FAM-MGS** | 4331182 |
| *Rgs12* Rn00571027_m1 | **FAM-MGS** | 4331182 |
| *Scrn1* Rn01410143_m1 | **FAM-MGS** | 4331182 |
| *Snx2* Rn01491606_m1 | **FAM-MGS** | 4351372 |
| *Vcpip1* Rn00557928_m1 | **FAM-MGS** | 4331182 |
| *Wdr1* Rn01453183_m1 | **FAM-MGS** | 4331182 |
| *Actb* Rn00667869_m1 | **VIC-MGS** | 4448489 |
| *Gapdh* Rn01775763_g1 | **VIC-MGS** | 4448489 |

**Supplemental Table 3: List of antibodies used:**

| **Primary Antibody Name** | **Company** | **Catalogue number** | **Dilution** |
| --- | --- | --- | --- |
| Ogt Rabbit mAb | Cell Signaling | 24083 | 1:1000 |
| Rgs12 Rabbit polyclonal Ab | LSBio | LS-C805328 | 1:1000 |
| Scrn1 Mouse mAb | LSBio | LS-C338451 | 1:1000 |
| Snx2 Rabbit polyclonal Ab | ThermoFisher Scientific | PA5-83367 | 1:1000 |
| Vcpip1 (VCIP) Rabbit polyclonal Ab | Abcam | Ab97812 | 1:1000 |

| **Secondary Antibody Name** | **Company** | **Catalog number** | **Dilution** |
| --- | --- | --- | --- |
| Anti-mouse IgG (H+L) HRP conjugated | Promega | W4021 | 1:10000 |
| Anti-rabbit IgG (H+L) HRP conjugated | Promega | W4011 | 1:10000 |

**Supplemental Figure 1: Body weight of animals.** Weight was recorded before and after surgery, as well as during self-administration.


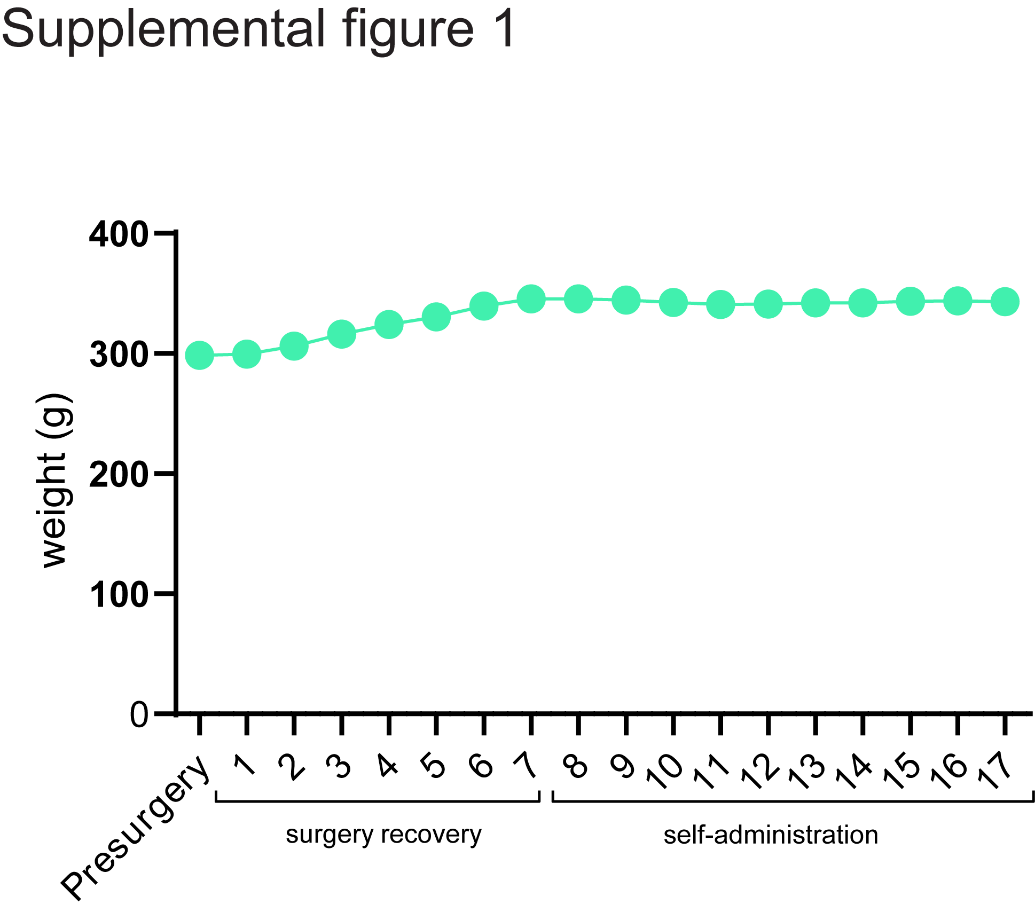


**Supplemental Figure 2: Animals in 2 and 21D abstinence groups display similar self-administration behavior.** Displayed are the average number of active lever presses (A-B) and number of infusions (C-D) over the last three days of self-administration of 0.075 mg/kg/infusion heroin (A,C) or 0.03 mg/kg/infusion (B,D). Error ± S.E.M. N=12/group.


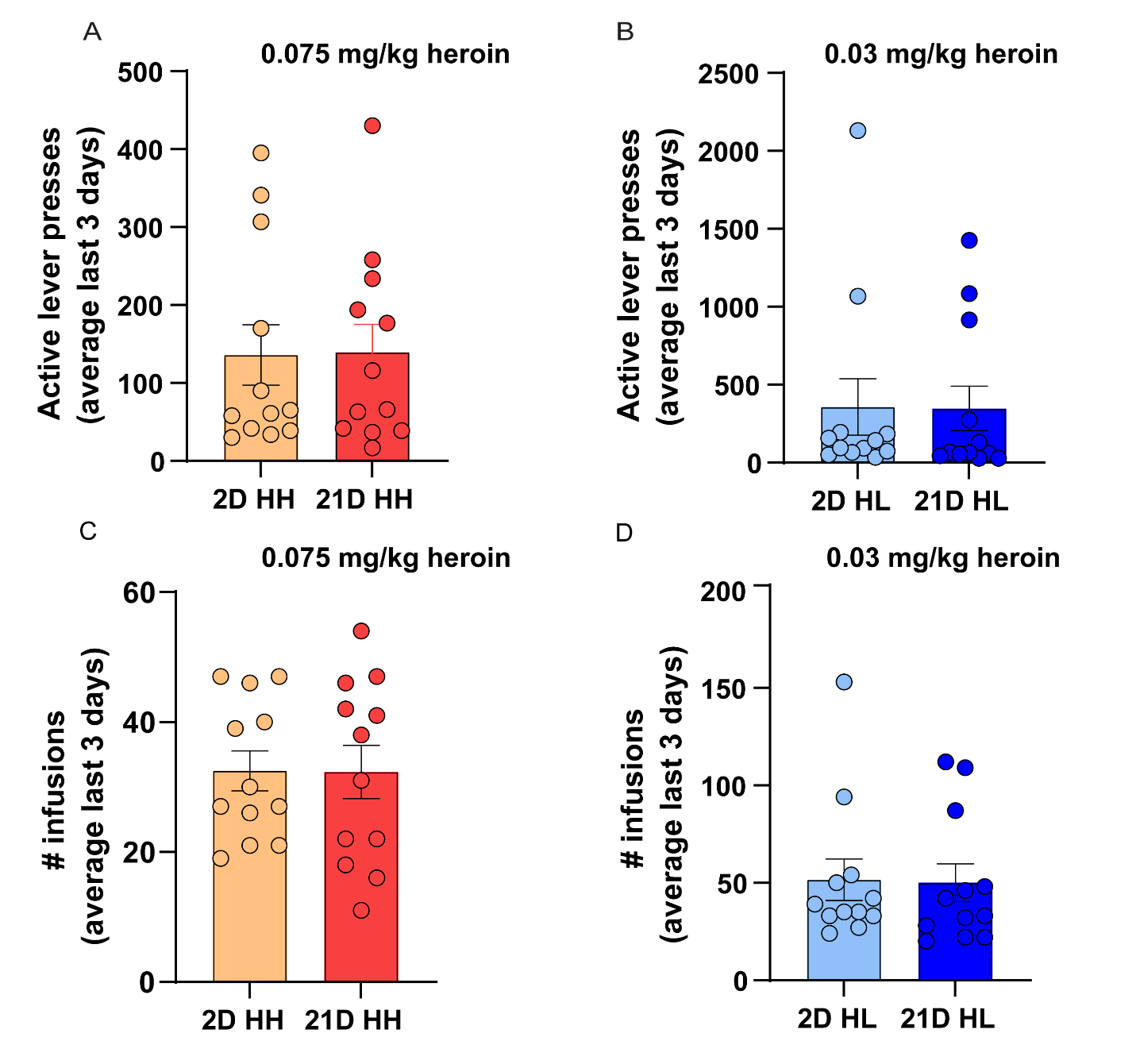


**Supplemental Figure 3: Measurement of miR-485-5p in the nucleus accumbens following forced abstinence.** Expression levels of mir-485-5p, obtained with qPCR, in the nucleus accumbens of rats that underwent SA of 0.075mg/kg/infusion heroin (HH) followed by 2D or 21D forced abstinence, or age-matched drug-naïve (N) controls. Error ± S.E.M. N=4-8/group.

**
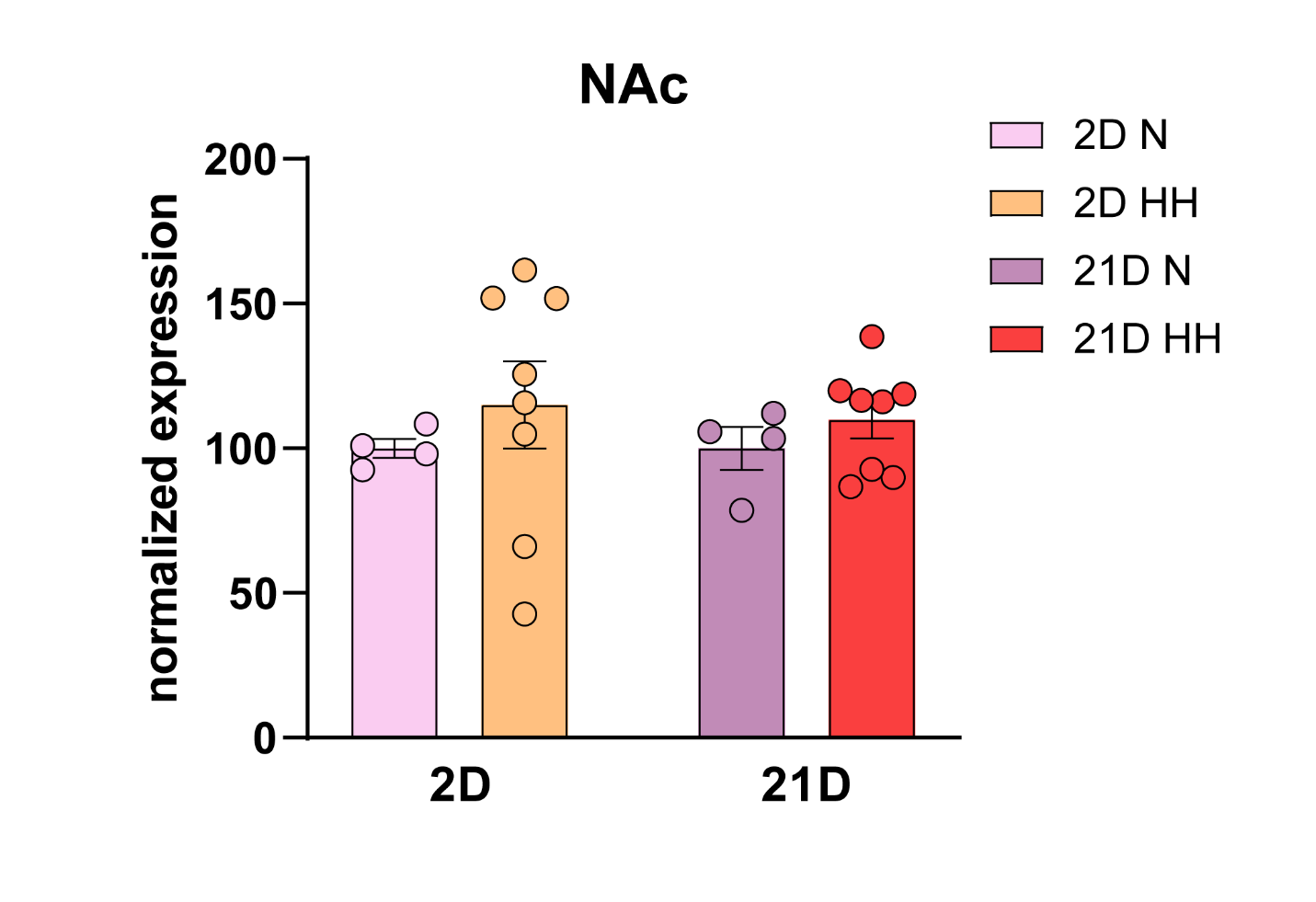
**

**Supplemental Figure 4: Validation of tools to manipulate miR-485-5p in vivo.** (A-B) In-vivo validation of miR-485-5p overexpression in the OFC of drug-naïve animals with miR-485-5p mimic (A) or a virus that overexpresses miR-485 (B). (C) Molecular validation of miR-485 overexpression efficacy in animals that underwent behavioral studies. Animals were euthanized right after the second relapse test. (D) In-vivo validation of miR-485-5p knockdown in the OFC of drug-naïve animals with the miR-485-5p inhibitor. Error ± S.E.M. *p<0.05; **p<0.01; ***p<0.001. N=3-4/group.


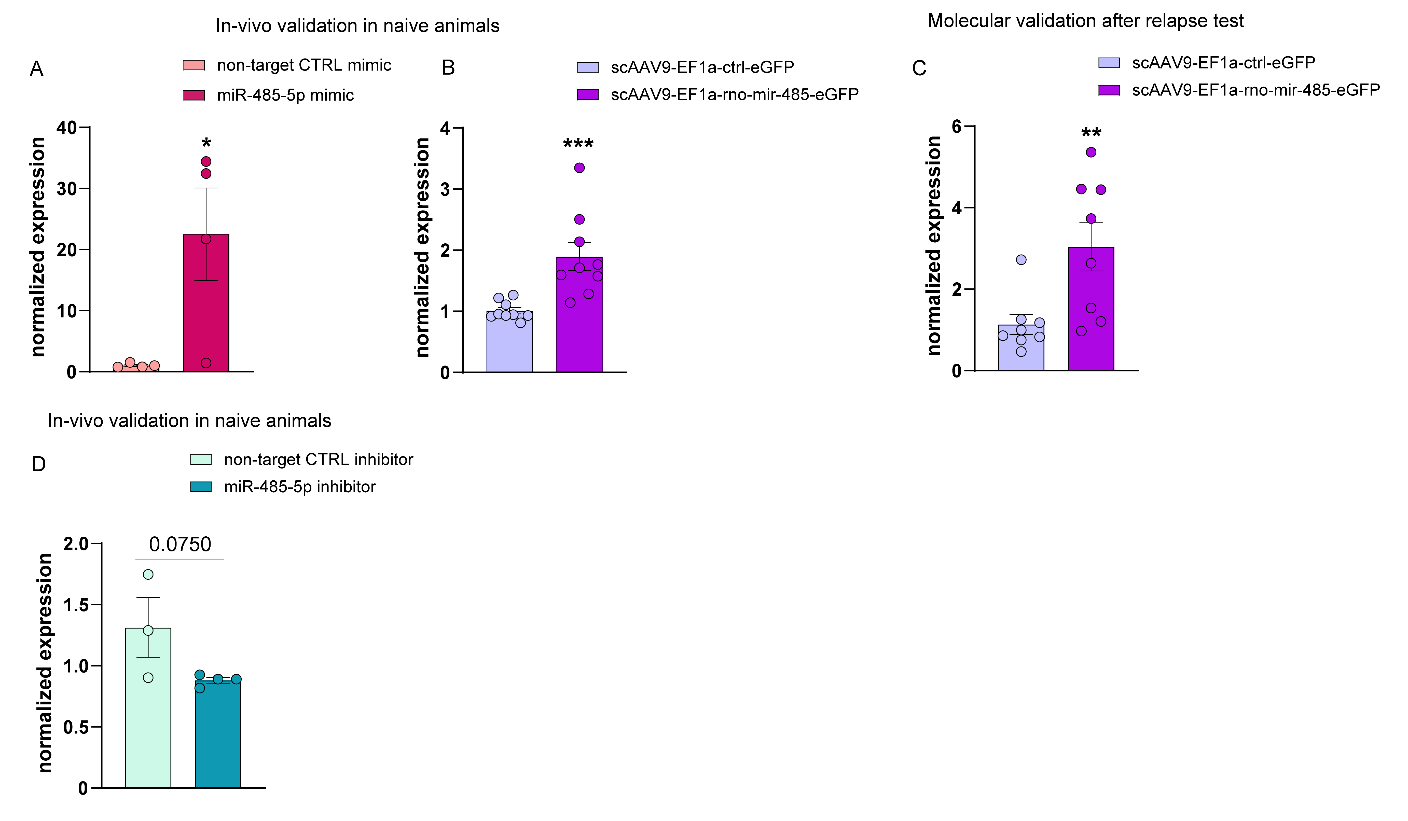


**Supplemental Figure 5: Measurement of miR-485-5p putative target genes in animals with miR-485-5p inhibition.** (A) *Drg1* expression in drug-naïve animals that received OFC infusion of scAAV9-EF1a-rno-485-eGFP or a scrambled CTRL virus. (B) *Drg1* expression in drug-naïve animals after OFC infusion of the miR-485-5p inhibitor or nontargeting control. (C) *Ogt* expression in drug-naïve animals after OFC infusion of the miR-485-5p mimic or nontargeting control. (D) *Ogt* expression in drug-naïve animals that received OFC infusion of scAAV9-EF1a-rno-485-eGFP or a scrambled CTRL virus. (E) *Ogt* expression in drug-naïve animals after OFC infusion of the miR-485-5p inhibitor or nontargeting control. (F) *Rgs12* expression in drug-naïve animals after OFC infusion of the miR-485-5p mimic or nontargeting control. (G) *Rgs12* expression in drug-naïve animals that received OFC infusion of scAAV9-EF1a-rno-485-eGFP or a scrambled CTRL virus. (H) *Rgs12* expression in drug-naïve animals after OFC infusion of the miR-485-5p inhibitor or nontargeting control Error ± S.E.M. *p<0.05; **p<0.0. N=4-5/group.


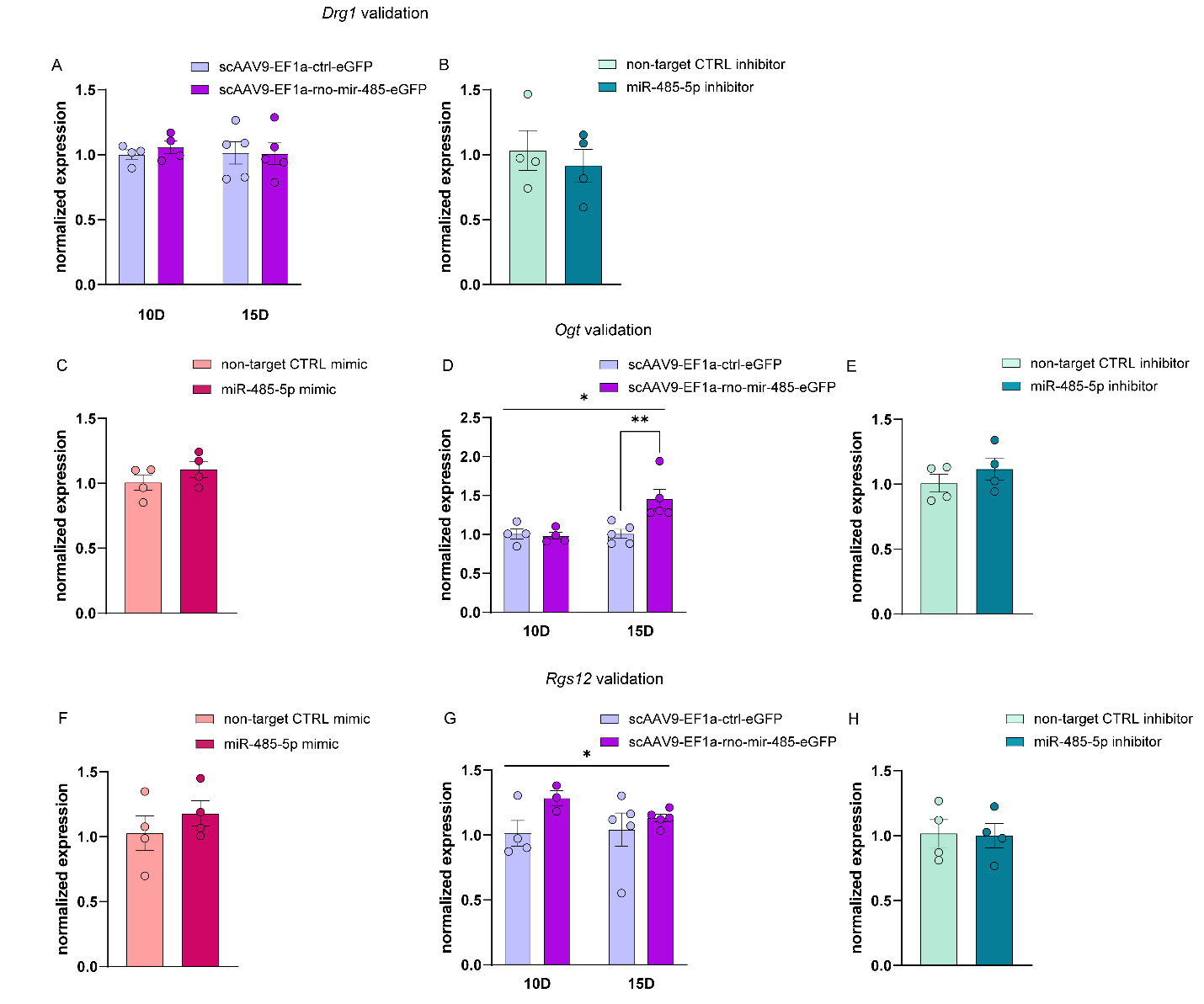


**Supplemental Figure 6: Overexpression of OFC mir-485-5p accelerates inhibition of active lever responding during relapse tests.** Survival curves of the percent of animals that reduced their active lever responding to 25% of responses made during heroin SA at relapse test 1 (A) or relapse test 2 (B). *p<0.05.


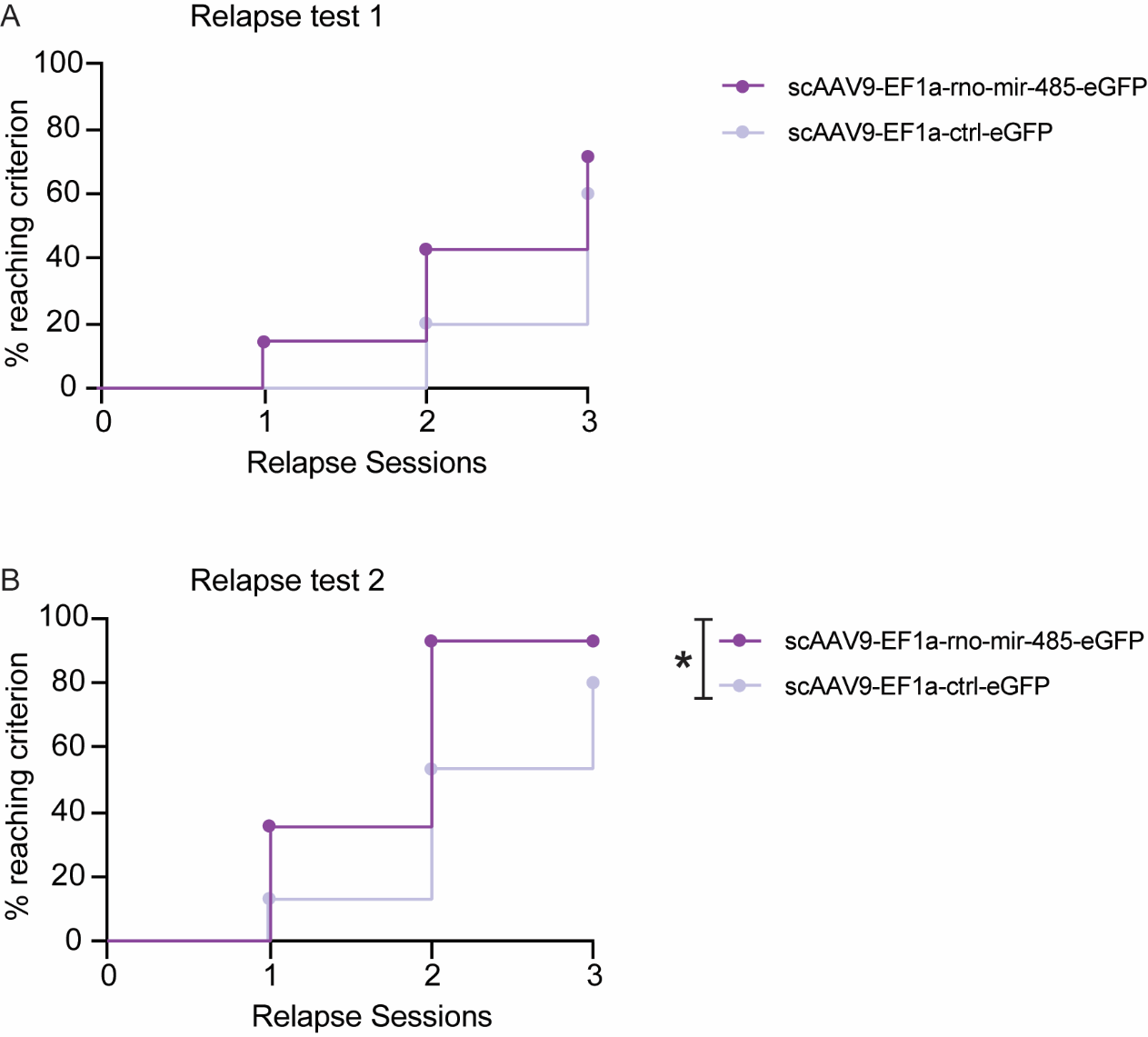


**Supplemental Table 4: Convergence of miRNAs and proteins associated with incubation of heroin craving behavior.**

**Supplemental western blot images:**

Ogt


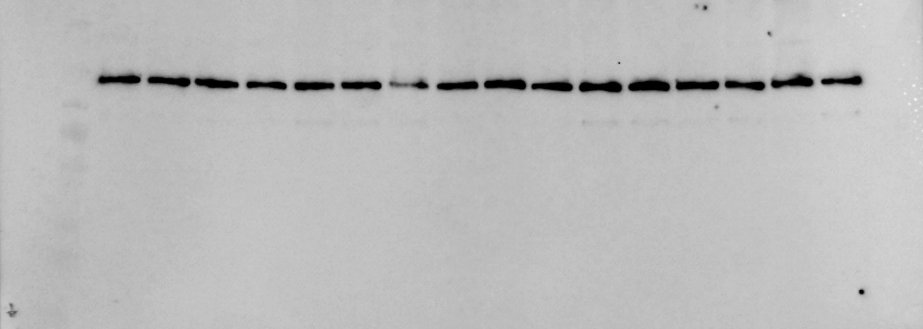


Gapdh

**
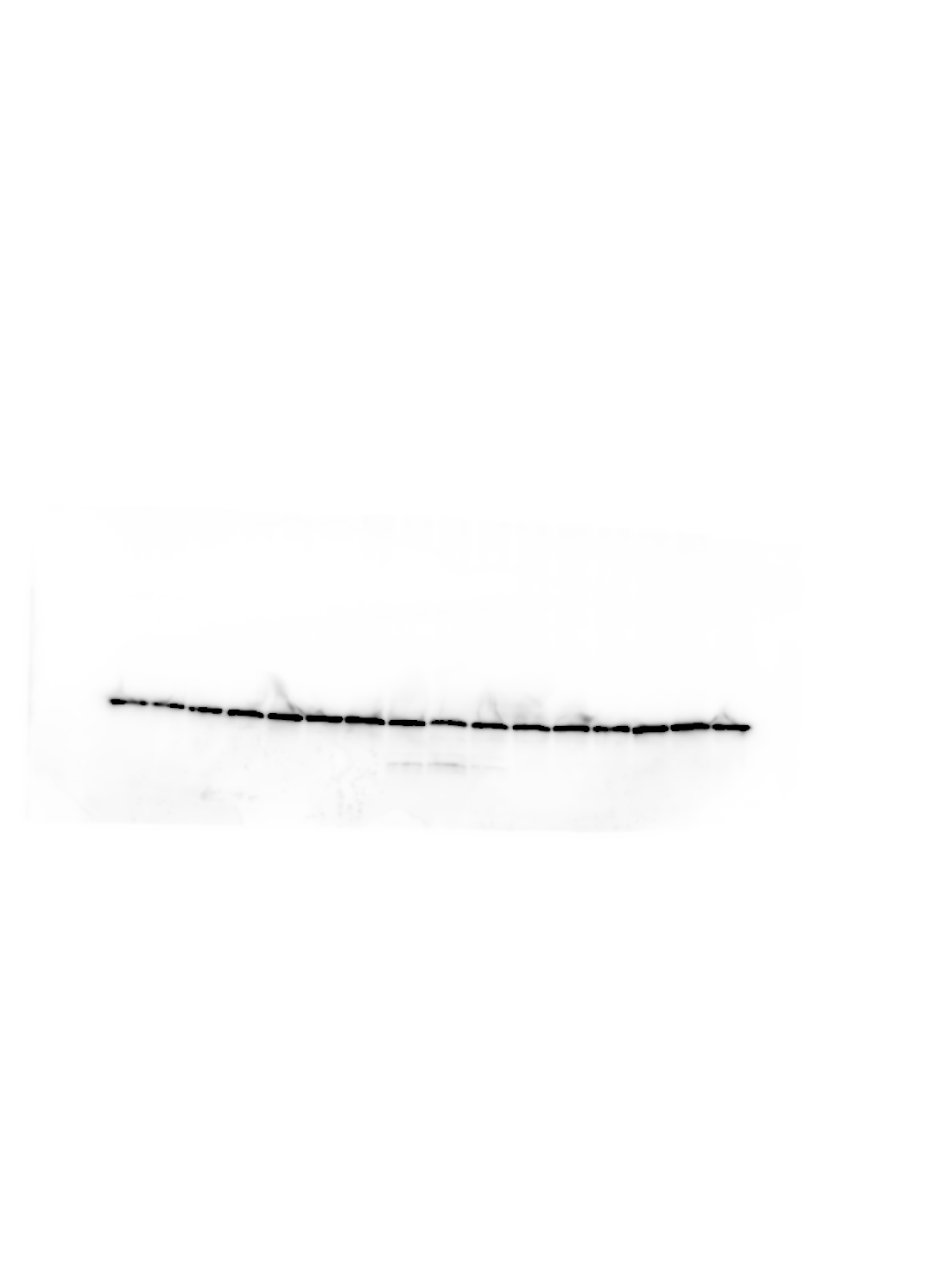
**

Rgs12





Total protein stain


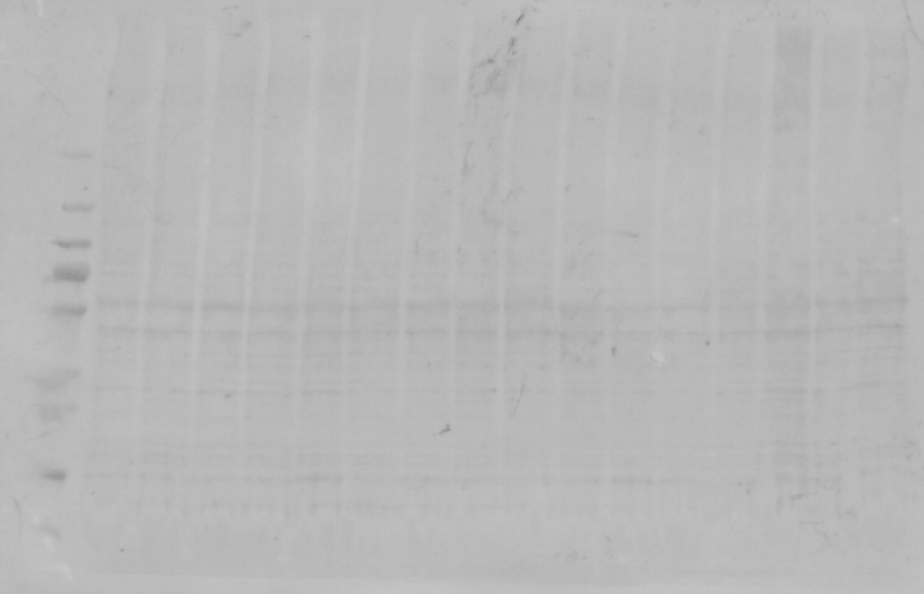


Scrn1

**
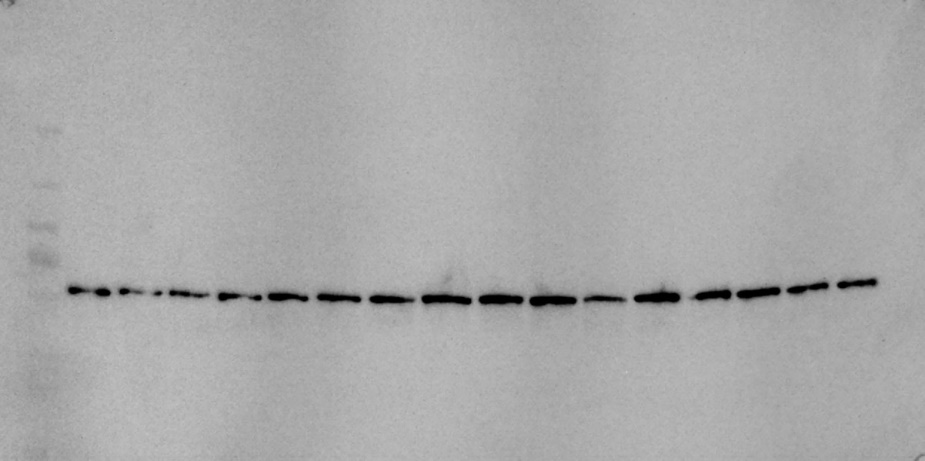
**

Total protein stain

**
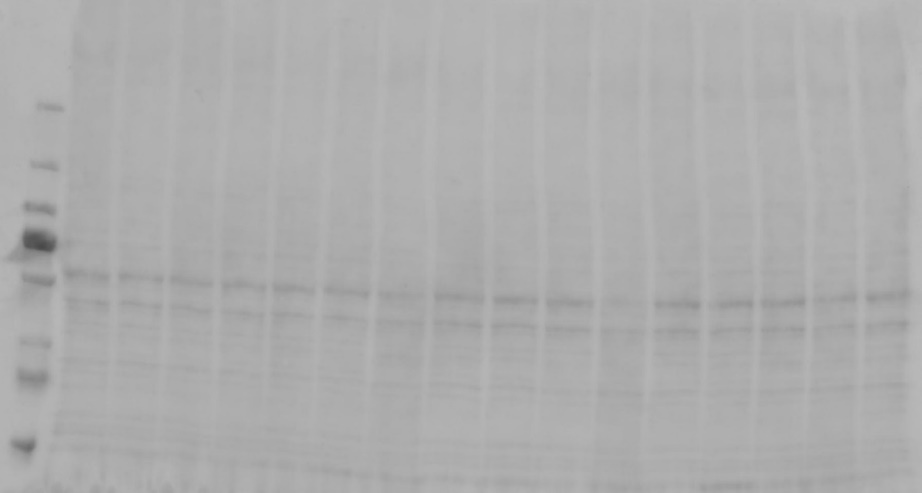
**

Snx2

**
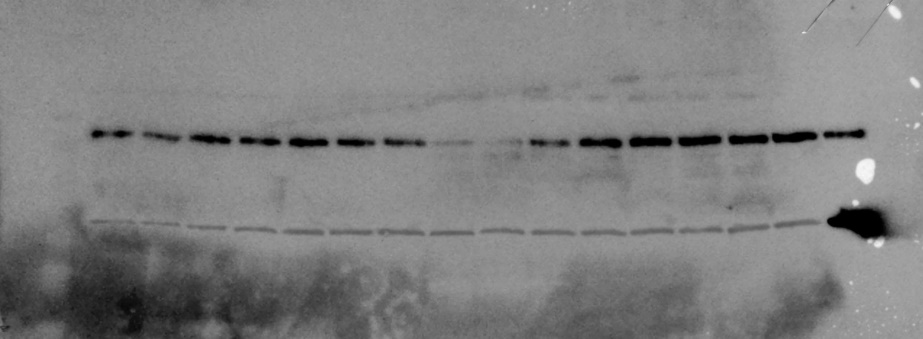
**

Gapdh

**
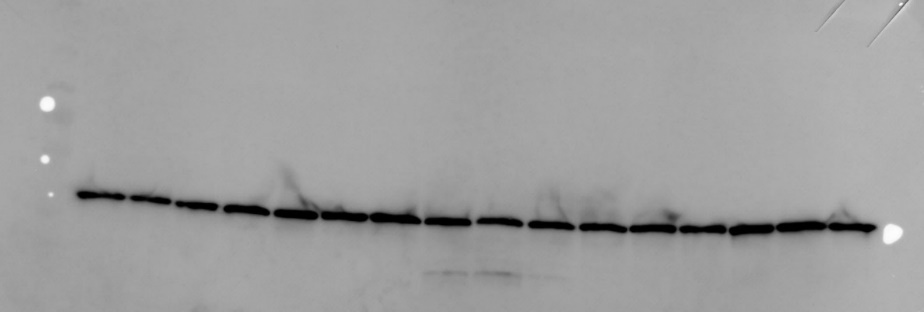
**

Vcpip1


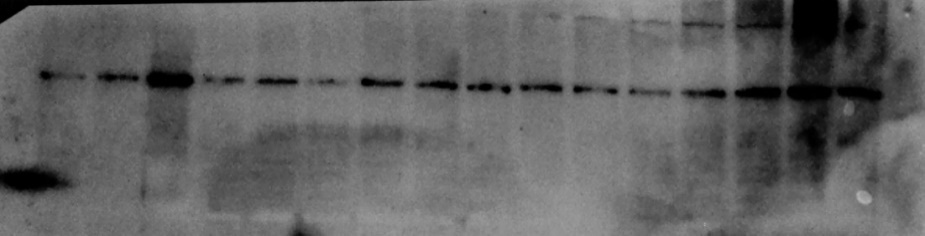


Gapdh


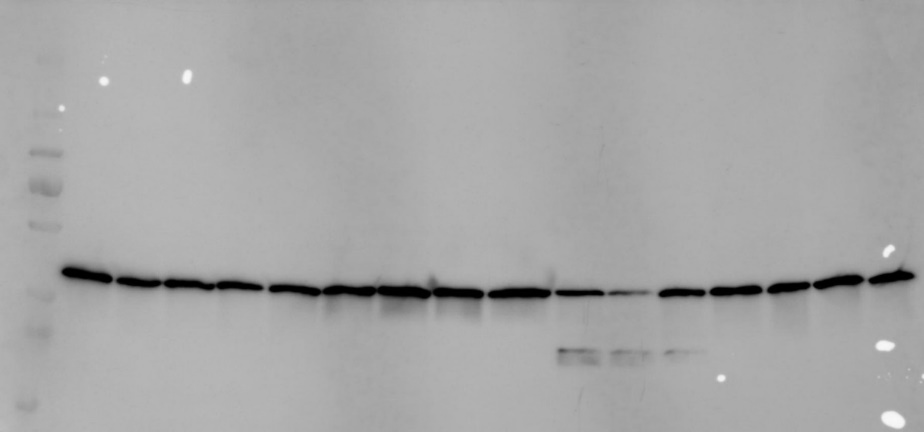


References:

1. Zanda, M.T., G. Floris, and S.E. Sillivan, *Author Correction: Drug-associated cues and drug dosage contribute to increased opioid seeking after abstinence.* Sci Rep, 2021. **11**(1): p. 23910.

2. Funk, D., et al., *Role of Central Amygdala Neuronal Ensembles in Incubation of Nicotine Craving.* J Neurosci, 2016. **36**(33): p. 8612-23.

3. Sillivan, S.E., et al., *Bioinformatic analysis of long-lasting transcriptional and translational changes in the basolateral amygdala following acute stress.* PLoS One, 2019. **14**(1): p. e0209846.

4. Langmead, B., et al., *Ultrafast and memory-efficient alignment of short DNA sequences to the human genome.* Genome Biol, 2009. **10**(3): p. R25.

5. Love, M.I., W. Huber, and S. Anders, *Moderated estimation of fold change and dispersion for RNA-seq data with DESeq2.* Genome Biol, 2014. **15**(12): p. 550.

6. Huang, d.W., B.T. Sherman, and R.A. Lempicki, *Systematic and integrative analysis of large gene lists using DAVID bioinformatics resources.* Nat Protoc, 2009. **4**(1): p. 44-57.

7. Sherman, B.T., et al., *DAVID: a web server for functional enrichment analysis and functional annotation of gene lists (2021 update).* Nucleic Acids Res, 2022.

8. Sillivan, S.E., et al., *MicroRNA regulation of persistent stress-enhanced memory.* Mol Psychiatry, 2019.

9. Rumbaugh, G., et al., *Pharmacological Selectivity Within Class I Histone Deacetylases Predicts Effects on Synaptic Function and Memory Rescue.* Neuropsychopharmacology, 2015. **40**(10): p. 2307-16.
